# Comprehensive Assignment of Roles for *Salmonella* Typhimurium Genes in Intestinal Colonization of Food-Producing Animals

**DOI:** 10.1371/journal.pgen.1003456

**Published:** 2013-04-18

**Authors:** Roy R. Chaudhuri, Eirwen Morgan, Sarah E. Peters, Stephen J. Pleasance, Debra L. Hudson, Holly M. Davies, Jinhong Wang, Pauline M. van Diemen, Anthony M. Buckley, Alison J. Bowen, Gillian D. Pullinger, Daniel J. Turner, Gemma C. Langridge, A. Keith Turner, Julian Parkhill, Ian G. Charles, Duncan J. Maskell, Mark P. Stevens

**Affiliations:** 1 Department of Veterinary Medicine, University of Cambridge, Cambridge, United Kingdom; 2 Enteric Bacterial Pathogens Laboratory, Institute for Animal Health, Compton, Berkshire, United Kingdom; 3 The Wellcome Trust Sanger Institute, Wellcome Trust Genome Campus, Hinxton, United Kingdom; 4 The ithree institute, University of Technology Sydney, Broadway, Australia; Uppsala University, Sweden

## Abstract

Chickens, pigs, and cattle are key reservoirs of *Salmonella enterica*, a foodborne pathogen of worldwide importance. Though a decade has elapsed since publication of the first *Salmonella* genome, thousands of genes remain of hypothetical or unknown function, and the basis of colonization of reservoir hosts is ill-defined. Moreover, previous surveys of the role of *Salmonella* genes *in vivo* have focused on systemic virulence in murine typhoid models, and the genetic basis of intestinal persistence and thus zoonotic transmission have received little study. We therefore screened pools of random insertion mutants of *S. enterica* serovar Typhimurium in chickens, pigs, and cattle by transposon-directed insertion-site sequencing (TraDIS). The identity and relative fitness in each host of 7,702 mutants was simultaneously assigned by massively parallel sequencing of transposon-flanking regions. Phenotypes were assigned to 2,715 different genes, providing a phenotype–genotype map of unprecedented resolution. The data are self-consistent in that multiple independent mutations in a given gene or pathway were observed to exert a similar fitness cost. Phenotypes were further validated by screening defined null mutants in chickens. Our data indicate that a core set of genes is required for infection of all three host species, and smaller sets of genes may mediate persistence in specific hosts. By assigning roles to thousands of *Salmonella* genes in key reservoir hosts, our data facilitate systems approaches to understand pathogenesis and the rational design of novel cross-protective vaccines and inhibitors. Moreover, by simultaneously assigning the genotype and phenotype of over 90% of mutants screened in complex pools, our data establish TraDIS as a powerful tool to apply rich functional annotation to microbial genomes with minimal animal use.

## Author Summary

*Salmonella* Typhimurium is a major cause of human diarrhoeal infections, usually acquired from chickens, pigs, cattle, or their products. To understand the basis of persistence and pathogenesis in these reservoir hosts, and to inform the design of novel vaccines and treatments, we generated a library of 7,702 *S.* Typhimurium mutants, each bearing an insertion at a random position in the genome. Using DNA sequencing, we identified the disrupted gene in each mutant and determined its relative abundance in a laboratory culture and after experimental infection of mice, chickens, pigs, and cattle. The method allowed large numbers of mutants to be investigated simultaneously, drastically reducing the number of animals required to perform a comprehensive screen. We identified mutants that grow in culture but do not survive in one or more of the animals. The genes disrupted in these mutants are inferred to be important for the infection process. Most of these genes were required in all three food-producing animals, but smaller subsets of genes may mediate persistence in a specific host species. The data provide the most comprehensive map of virulence-associated genes for any bacterial pathogen in natural hosts and are highly relevant for the design of control strategies.

## Introduction

*Salmonella enterica* is a facultative intracellular pathogen of worldwide importance, associated with c. 21.7 million cases of systemic typhoid fever and 93.8 million cases of non-typhoidal gastroenteritis in humans each year [1], [2]. Around 86% of human cases of non-typhoidal salmonellosis are the result of food-borne infections [2], and chickens, pigs and cattle are key reservoirs of infection [3]. The major *S. enterica* subspecies *enterica* encompasses a wide variety of serovars. Some of these, such as *S.* Typhimurium and *S.* Enteritidis, exhibit a wide host range, whereas others such as *S.* Typhi are largely restricted to a single host species. The molecular basis of the host- and tissue-tropism of *S. enterica* has long eluded researchers and there has been a disproportionate emphasis on the basis of *Salmonella* persistence and pathogenesis in murine models of colitis and typhoid fever. Comparative analyses of whole genome sequences have associated host-restriction of *S. enterica* serovars with gene decay. Interpretation of the impact of variation in the repertoire, sequence or expression of *S. enterica* genes requires an understanding of the roles of those genes in relevant hosts. Random transposon insertion mutants have been screened individually in chickens [4], but high-throughput simultaneous analysis of mutant phenotypes was made possible by the advent of signature-tagged mutagenesis (STM) [5]. STM allows the survival of individual mutants within a pool to be assessed qualitatively through hybridization of a probe to a unique tag sequence within the transposon. Comparison of hybridization signals obtained from “input pools” of mutants grown *in vitro* with the those obtained from the same set of mutants screened for survival in a model of infection (“output pool”) allows attenuated mutants to be identified. The insertion sites can then be identified by subcloning and sequencing. Analysis of pools of signature-tagged *S.* Typhimurium mutants in mice [5] led to the discovery of *Salmonella* Pathogenicity Island (SPI)-2 [6], a gene cluster that encodes a type III secretion system (T3SS-2) that acts as a molecular syringe for the secretion of effector molecules and influences systemic virulence and intracellular survival [7]. A distinct T3SS, encoded by SPI-1, was already known to be essential for infection of mice via the oral route [8]. Subsequently, STM libraries constructed in a range of *Salmonella* serovars were examined for their ability to colonize multiple hosts [9]–[14]. Comparative analysis of a library of 1045 mutants in chickens, pigs and calves suggested that *S.* Typhimurium deploys both conserved- and host-specific virulence factors [10], [12]. Notably, SPI-1 and -2 were vital in intestinal colonization of calves and pigs and to a lesser extent chickens [10], [12], yet a Type I protein secretion system encoded by SPI-4 appeared to influence infection of calves, but not chickens [12] or pigs [10].

**Figure 1:**
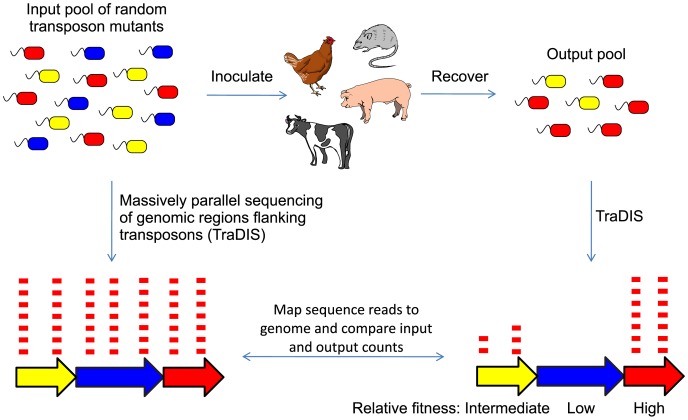
Experimental strategy for TraDIS mutant screens. An input pool of random transposon insertion mutants is generated, and used to inoculate experimental animals. Output pools of bacteria that are capable of survival and growth in each host are isolated from an appropriate tissue. Massively parallel sequencing of the regions flanking each transposon allow the disrupted genes to be identified, and comparison of the sequence counts derived from the input and output pools allows the relative fitness of each mutant to be assessed.

Although STM has provided valuable insights into *Salmonella* pathogenesis, the technique is limited by the number of unique tags available, and the time and effort required to construct the library and identify attenuating mutations. Moreover, only negatively-selected mutants tend to be investigated and subjective judgments are used to compare signal intensities relative to the input and to other screened mutants. Transposon-directed insertion-site sequencing (TraDIS) [15], one of a new generation of STM-like techniques, addresses some of these limitations. TraDIS exploits Illumina sequencing [16] to obtain the sequence of the genomic region flanking each transposon. The massively parallel nature of the sequencing permits comparison of the number of specific reads derived from the input pools and the output pools after animal infection, providing a numerical measure of the extent to which mutants were negatively- or positively-selected during colonization (see Figure 1). TraDIS-like sequencing methods have been used to identify the essential gene complement of *S.* Typhi [15] and *Streptococcus pneumoniae* [17], genes involved in virulence of *Haemophilus influenzae* [18] and *S. pneumoniae* [19] in mice, and genes required for survival of the symbiont *Bacteroides thetaiotaomicron* in the murine gut [20]. Here we apply TraDIS to simultaneously assign the genotype and relative fitness of 7702 distinct *S.* Typhimurium mutants during intestinal colonization of chickens, pigs and cattle, providing highly relevant data for the control of zoonotic and animal salmonellosis.

## Results

### Quantitative assessment of mutant fitness

To validate the quantitative nature of TraDIS, we applied it to investigate pools of Mu and mini-Tn*5* mutants of *S.* Typhimurium strain SL1344 before and after intravenous infection of BALB/c mice (see Text S1). These mutant pools had been characterized previously using transposon-mediated differential hybridization (TMDH) [21], a microarray-based method that relies on hybridization of run-off transcripts arising from transposon-encoded T7 and SP6 promoters to high-density oligonucleotide arrays. In total, using TraDIS, 9792 distinct transposon insertions (4992 Mu and 4800 Tn*5*) were unambiguously mapped at the level of the single nucleotide to the SL1344 genome, providing relative fitness scores for 94.4% of the 10368 mutants screened, This is likely to be an underestimate of the performance of TraDIS, since it is likely that there were siblings of mutants with mapped insertions within the pool of 10368 mutants. The fitness scores assigned by TraDIS are defined as the log_2_-fold change in the number of sequence reads obtained across the boundaries of each transposon insertion between the input and output pools (see Materials and Methods), and were significantly correlated with the existing TMDH data (Figure S1; *P*<2.2×10^−16^), verifying the quantitative nature of TraDIS. TraDIS allowed identification of a number of mutants missed by TMDH (see Text S1), and provided finer mapping of insertion sites than can be achieved by hybridization of transposon-flanking sequences to tiling arrays, extending the conclusions of the earlier study and demonstrating the superiority of the TraDIS approach.

### Assessment of mutant fitness in food animals

**Figure 2:**
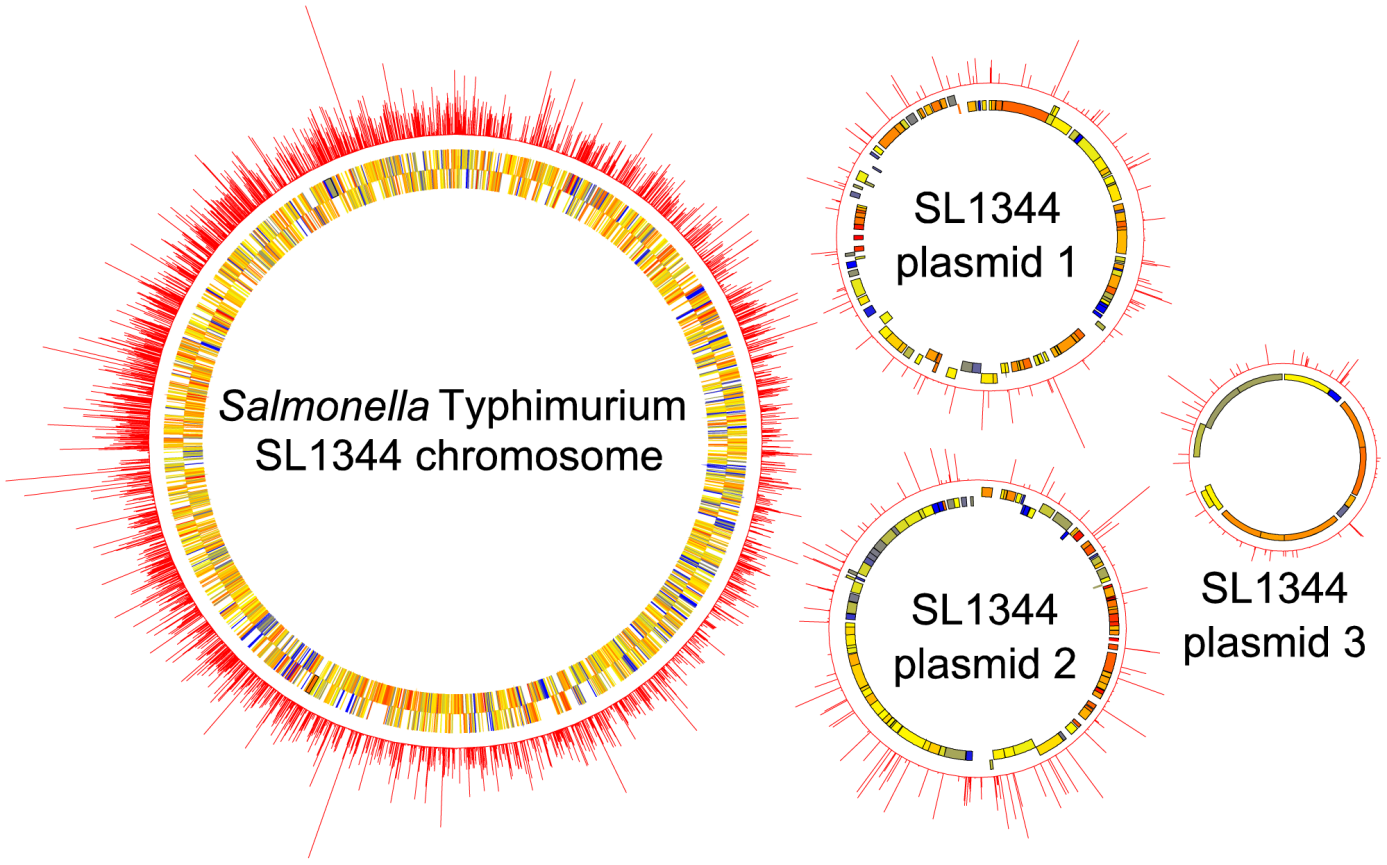
Circular diagrams of the *S.* Typhimurium SL1344 chromosome and plasmids (not to scale), showing the near random distribution and high density of transposon insertions mapped by TraDIS. The inner two rings indicate the positions of annotated genes, coloured according to their GC content (blue = low, yellow = intermediate, red = high). The outer ring indicates the number of transposon-flanking sequence reads obtained at each position, with peaks corresponding to the presence of a transposon insertion.

Though useful, previous attempts to assign comprehensively the role of *S.* Typhimurium genes relied on parenteral infection of atypically susceptible mice [21]–[23], and do not reflect the roles of *Salmonella* genes during intestinal colonization of food-producing animals infected via the natural oral route. To identify genes relevant to colonization of animal reservoirs and therefore zoonosis, we generated a library of 8550 mini-Tn*5* mutants of *S.* Typhimurium strain ST4/74 and applied TraDIS to assess the survival of these mutants during oral infection of chickens, pigs and calves. Pools of 475 mutants were screened in individual pigs and calves and pools of 95 mutants were screened in duplicate chickens. Pilot studies in which selected pools had been repeatedly screened in each species indicated the reliable negative selection of the same mutants at these pool complexities in the absence of stochastic loss that may be due to population bottlenecks (data not shown). TraDIS mapped 7702 distinct insertions to the nucleotide level, representing at least 90.1% of the mutants screened, and demonstrated the random distribution of the transposon insertions around the genome (see Figure 2), which disrupt 2715 different genes. TraDIS analysis revealed evidence of random drop-out of mutants from the pools in one chicken, two pig and two calf experiments, likely owing to recovery of output pools of an inadequate size, and data from these animals were omitted from subsequent analysis (see Text S1).

**Figure 3:**
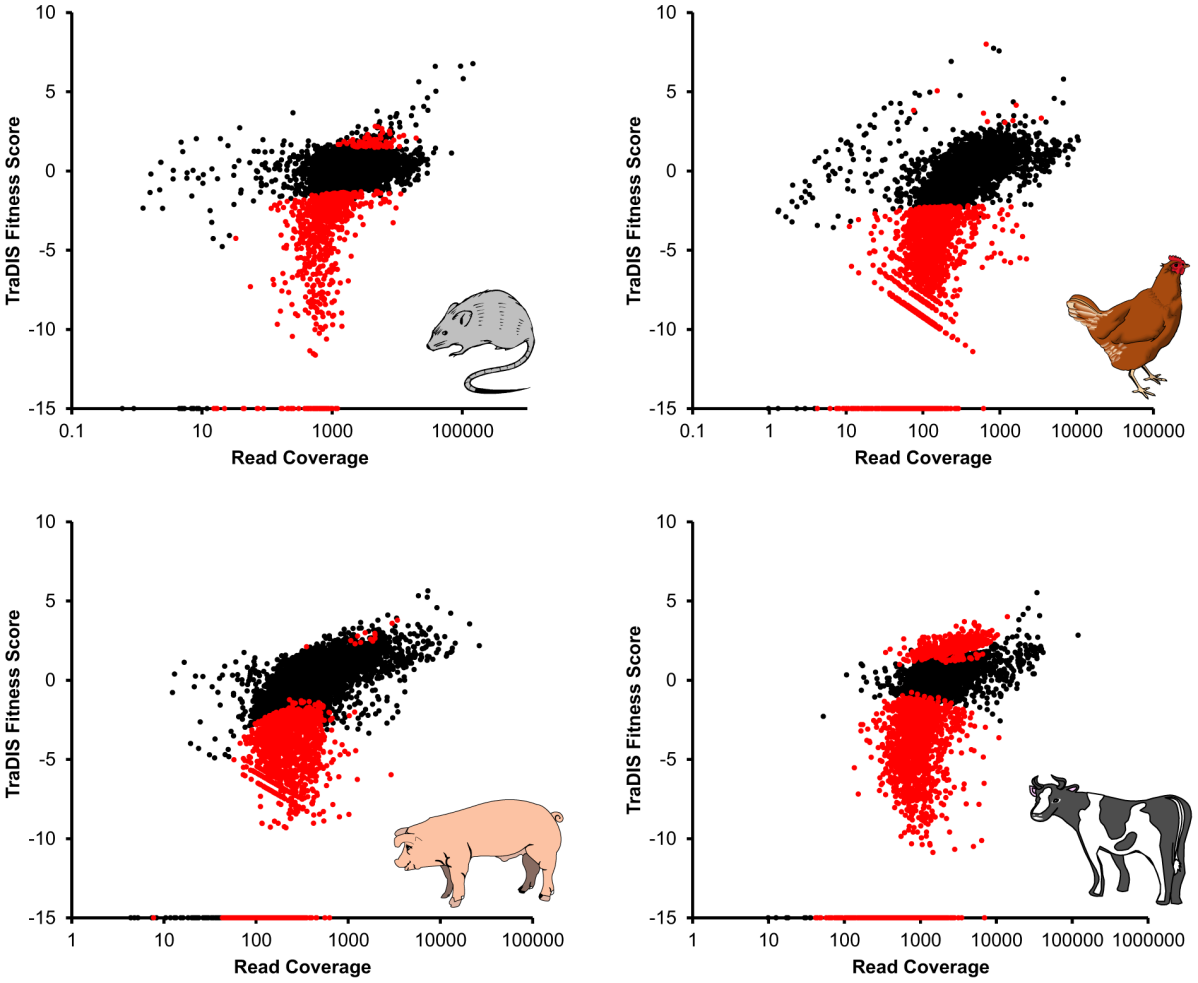
Relative fitness scores (defined as log_2_ fold changes in sequence read counts between input and output pools) for each transposon mutant, plotted against average read coverage (equivalent to an M/A plot, commonly used to display microarray data) for each of the four host species. Mutants which showed a significant change in abundance in the output relative to the input are highlighted in red. Mutants which had no reads in the output pool are assigned an arbitrary fitness score of −15.

TraDIS assignments of the insertion sites and fitness scores of mutants are listed in Table S1 (BALB/c mice dosed intravenously) and Table S2 (chickens, pigs and calves dosed orally). The raw TraDIS sequence data are available from the NCBI Short Read Archive (accession numbers ERA000172 and ERP000286). To facilitate exploration of the TraDIS data, a user-friendly online genome browser was constructed with which the insertion site and fitness score can be viewed in the context of the linear genome, GC content, transcription start sites and existing annotation (http://www-tradis.vet.cam.ac.uk). Figure 3 shows the fitness scores obtained by TraDIS analysis of *S.* Typhimurium mutants screened in mice, chickens, pigs and calves, plotted against read coverage (equivalent to the “M/A” plots commonly used to display microarray data). The proportion of significantly attenuated mutants identified during intestinal colonization of food-producing animals was greater than in the murine typhoid model. In each host, a large proportion of mutations did not exert a strong negative or positive effect, indicating that a high number of accessory or redundant functions exist. *P* values were estimated using the available biological replicates (all pools were screened in duplicate in chickens, 2 pools were screened in duplicate calves and 3 pools were screened in triplicate pigs), and attenuated mutants were defined as those with a negative fitness score and *P*≤0.05.

To summarize the dataset further, genes were scored as potentially important in colonization if they were disrupted in at least one significantly attenuated mutant in any of the four host species. This enables comparisons between datasets derived from different transposon libraries (such as the mouse and chicken/pig/cattle datasets), and visualization of the data in comparison with other genome-wide datasets. For some genes, insertions at different subgenic locations can have divergent effects on the encoded protein resulting in contrasting fitness scores, so it is important to consider the genetic context of each individual transposon when interpreting the TraDIS data in detail. This is true for all transposon-based mutant screens, although earlier technologies such as STM and TMDH lacked sufficient resolution to permit such considerations. We recommend the use of the TraDIS browser (http://www-tradis.vet.cam.ac.uk) to assist interpretation of our data in the context of the genome annotation.

**Figure 4:**
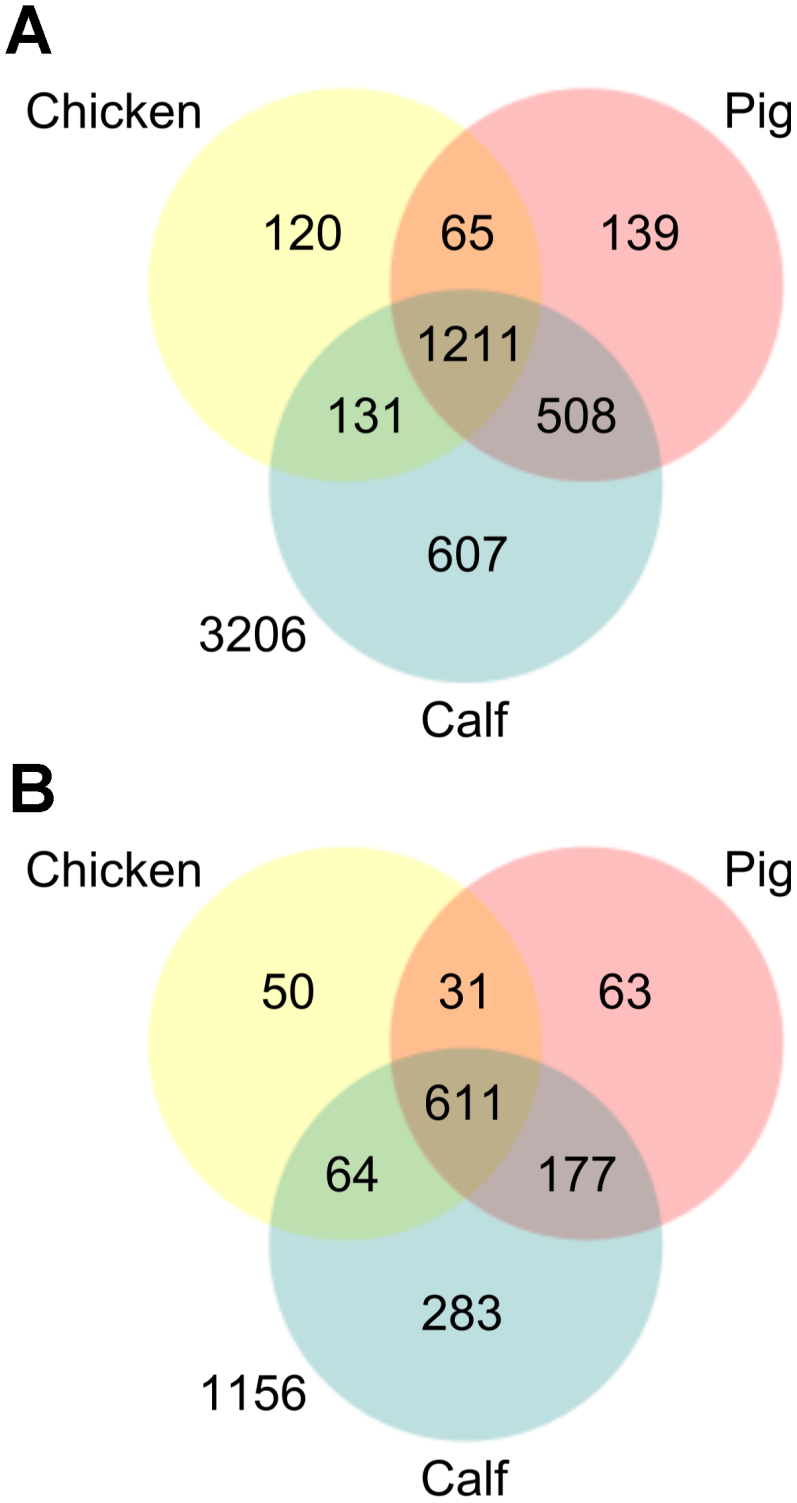
Venn diagrams showing the attenutation phenotypes observed in chickens, pigs, and cattle using TraDIS. A) the numbers of transposon mutants which were significantly attenuated in each host B) the numbers of genes which were disrupted in the TraDIS mutant library, and which are potentially important for colonization (*i.e.* they had at least one significantly attenuated mutant) in each of the three reservoir hosts.

Fitness scores were obtained for 3194 distinct genes disrupted by transposon insertions in the mouse screen, and 2715 genes in the chicken, pig and calf screens, of which fitness score existed for 2435 genes in all three food-producing animals. Fitness scores were available from all four hosts for 1935 genes, of which 1069 had a significantly attenuated mutant in at least one host. Venn diagrams showing the numbers of significantly attenuated mutants, and the numbers of genes potentially important for colonization in chickens, pigs and cattle are shown in Figure 4. A further Venn diagram, combining the chicken/pig/cattle and mouse datasets, is available in Figure S2, and Figure S3 shows a comparison between the chicken, pig and cattle TraDIS data, and the data obtained from equivalent mutants in the earlier STM screens [10], [12]. A table of all genes disrupted by a transposon insertion, indicating if any of the mutants in each gene was significantly attenuated, is available in Table S3. To facilitate exploration of the TraDIS data, custom files have been prepared that allow proteins in KEGG metabolic pathway diagrams [24] to be coloured blue if an attenuated mutant was found in the encoding gene, or red if the gene was mutated but no significant attenuation was observed. The KEGG colour files are available from http://www-tradis.vet.cam.ac.uk. As an example, Figure S4 shows the effect of mutations affecting multiple steps in chorismate biosynthesis, which is known to influence persistence of *S.* Typhimurium *in vivo*. It is evident that mutations affecting sequential steps in the pathway are attenuating, with just two exceptions: the initial condensation of D-erythrose-4-phosphate and phosphoenolpyruvate into 3-deoxy-D-arabino-heptulosonate-7-phosphate, and the conversion of shikimate to shikimate-3-phosphate, both of which can be catalyzed by the products of multiple genes (*aroFGH* and *aroKL*, respectively). Thus, TraDIS identifies pathways in which defects exert a common effect, but also reveals steps at which functional redundancy exists. During the analysis of the TraDIS data it became clear that many regions of the genome with low GC content are important for intestinal colonization of chickens, pigs and cattle (see Text S1 and Figure S5).

### Analysis of defined null mutants in chickens

Twelve genes were selected for further investigation based on the TraDIS data: *carB*, *clpB*, *ilvC*, *mig-14*, *pagN*, SL1344_0084 (STM0084), SL1344_4248 (STM4312), SL1344_3128 (STM3154), *trxA*, *virK*, *ytfL* and *zirT* (SL1344_1599). These targets were chosen based on their fitness scores (at least one mutant in each gene shows significant attenuation), and to include some genes with established roles in colonization in chickens (*clpB* [4]) or mice (*trxA* [25], *mig-14* [26], *virK* [27]), genes with a postulated role in colonization (*pagN* [28], SL1344_0084 [12]), the mouse anti-virulence factor *zirT* [29], genes demonstrating variable fitness scores (SL1344_0084, SL1344_3128 and *ytfL*) and genes that demonstrate putative host-specific effects on colonization in the TraDIS data (*clpB*, *ilvC* and *ytfL*). Each gene was inactivated separately by λRed recombinase-mediated integration of linear PCR amplicons by homologous recombination [30]. Mutant phenotypes were evaluated in groups of 3 chickens per mutant per time interval. For each mutant, competitive indices (CIs) were derived 4, 6 and 10 days post-inoculation of age-matched chickens with the kan^R^-tagged mutant and ST4/74 nal^R^ wild-type strain in a 1∶1 ratio (see Table 1).

**Table 1:** Competitive indices of defined null mutants of *S.* Typhimurium lacking candidate virulence-associated loci identified by TraDIS.

Mutation	Mean CI Day 4	Mean CI Day 6	Mean CI Day 10	Chicken TraDIS *Fitness* Score
*carB*	0.187 (0.0004)	0.171 (0.0016)	0.002 (<0.0001)	−5.43 to −15 (5/5)
*clpB*	0.047 (<0.0001)	0.001 (<0.0001)		−2.36 to −8.11 (4/4)
*ilvC*	0.294 (0.0026)	0.310 (0.0309)	0.104 (0.0001)	−2.05 to −3.38 (1/2)
*mig-14*	0.957 (0.9014)	0.469 (0.0159)	0.820 (0.2802)	−15 (1/1)
*pagN*	0.761 (0.2741)	0.613 (0.2588)	0.804 (0.1625)	−3.08 to −15 (4/4)
SL1344_0084	0.727 (0.0959)	1.062 (0.7933)	0.430 (0.0008)	−0.82 to −9.11 (6/7)
SL1344_4248	0.487 (0.0015)	1.152 (0.7458)	1.020 (0.8951)	−3.51 to −5.07 (4/4)
SL1344_3128	1.267 (0.3367)	1.001 (0.9963)	1.204 (0.7051)	−1.02 to −9.20 (7/9)
*trxA*	0.003 (<0.0001)	0.013 (<0.0001)		−3.58 to −8.21 (4/4)
*virK*	0.347 (0.0024)	0.638 (0.1336)	0.416 (0.0038)	−1.48 to −7.05 (3/4)
*ytfL*	0.184 (<0.0001)	0.175 (<0.0001)	0.033 (<0.0001)	0.44 to −4.38 (2/6)
*zirT* (SL1344_1599)	0.592 (0.0015)	0.581 (0.7458)	0.522 (0.8951)	−15 (1/1)

*P* values are shown in parentheses. The range of TraDIS fitness scores obtained in the chicken experiment is also shown for each gene, with the fraction of significantly attenuated mutants shown in parentheses.

With a single exception (SL1344_3128) all mutants were negatively-selected relative to the parent strain at day 4 post-inoculation, which corresponds to the time at which mutants were recovered for the TraDIS analysis. The difference in the mutant∶wild-type ratio was significantly different from the ratio in the inocula for 8 of the mutants at day 4. For a further 2 mutants, significant differences were detected at later time points. Taken together with comparisons to existing datasets for signature-tagged mutants of the same strain in the same animal models [10], [12] (see Text S1), the data strongly support evidence of attenuation detected by TraDIS. Variance from TraDIS fitness scores is likely to reflect differences in competition dynamics for a given mutant relative to co-screened wild-type or mutant bacteria. For one gene (SL1344_3128 ), no evidence was found of any attenuation of the defined mutant, which performed comparably to the wild-type at all time intervals. This gene was chosen for further investigation because its mutants exhibited a wide range of TraDIS fitness scores (−1.02 to −9.20). Interestingly, mutants in the gene cluster SL1344_3128-30 are predicted to be deficient in swarming motility [31], suggesting the possibility that such motility may be an occasional but not universal requirement for colonization.

## Discussion

### The genetic basis of intestinal colonization

The TraDIS dataset is a powerful resource for understanding intestinal colonization of a range of highly relevant hosts by *Salmonella*, and thus zoonotic transmission and animal disease. The data suggest that the definition of what constitutes a colonization gene is not straightforward, encompassing genes involved in metabolism, stress responses and transcriptional regulation, together with genes with well-established roles in virulence. The T3SSs encoded by SPI-1 and SPI-2 are both essential for infection in chickens, pigs and cattle, although there are some mutants within both regions that are not attenuated or exhibit a less pronounced phenotype in chickens. T3SSs allow the secretion of effector molecules into the host cytoplasm, these effectors being encoded both within SPI-1 and 2 and distally. Most of the known effector genes, including *sopA*, *sopB*, *sopE2*, *sipA*, *avrA*, *sipC*, *sseG*, *sseI*, *sifA*, *sseK1*, *pipB2* and *sopD2*, were identified by TraDIS as being important for infection of all three food-producing animals, although as with the T3SS structural genes, the phenotype was often less pronounced in chickens. Other T3SS effectors, including *sptP*, *slrP*, *gogB* and *sspH2*, could be disrupted without affecting colonization. Of these, *slrP* has been implicated as a host-specificity factor, essential for oral infection of mice but not required for calf infection [14]. Null mutants of *sptP* are not impaired in their interactions with cultured macrophages or epithelial cells [32]–[34], and *sspH2* mutants do not show any defect in vacuole-associated actin polymerization [35]. For both *sptP* and *sspH2*, the lack of phenotype was suggested to be due to functional redundancy amongst T3SS effectors.

There are also attenuated mutants that harbour insertions within the other recognized *Salmonella* pathogenicity islands [36]. In SPI-3, mutants in some genes (*mgtC*, *marT*, SL1344_3717 and SL1344_3721) were attenuated, with others (*misL*, *sugR*, *slsA* and *mgtB*) showing no attenuation. Interestingly, *marT*, which encodes a transcriptional regulator, is a pseudogene in *S.* Typhi, and restoring it reduces survival during infection of a human cell culture [37]. A role for *marT* in infection of chickens, pigs and cattle suggests a selection pressure for its retention in the *S.* Typhimurium genome. SPI-4 was previously thought to play a role in infection of cattle but not chickens or pigs, based on STM screens [10], [12]. TraDIS suggests a role for SPI-4 in all three species, although the phenotypes in chickens and pigs are more subtle than in calves, highlighting the increased sensitivity of TraDIS relative to STM. Some transposon insertions within the central highly repetitive region of *siiE* are tolerated in chickens and pigs, but are attenuated in cattle. Interestingly, *siiE* is split into two ORFs in both *S.* Typhi genome sequences, and part of the repetitive central region is absent from the genome of *S.* Paratyphi A. SiiE is secreted [38], indicating that *in trans* complementation by co-screened mutants does not obscure the identification of secreted colonization factors by TraDIS. All of the genes of the enteritis-associated SPI-5 that were disrupted by a transposon (*pipACD*, *sopB* and *orfX*) were required in all three species, although often with a milder phenotype in chickens. Several clusters of attenuated mutants were also found in the *Salmonella* chromosomal island (SCI, also known as SPI-6 in *S.* Typhi), including mutants in the hypothetical genes *sciJ*, *sciQ*, SL1344_0286A and *sciX*, the fimbrial subunit *safA* and its chaperone *safB*, the regulator *sinR*, SL1344_0301 (STM0305) which encodes a putative cytoplasmic protein, the *pagN* adhesin (STM0306) and *sciZ* (STM0307), a homologue of *Shigella virG*. Deletion of SCI affects invasion and virulence in a mouse intraperitoneal infection model [39], and the phenotype of a defined *safA* mutant has been confirmed in pigs [10].

Fimbriae play a well-established role in *Salmonella* attachment and intestinal colonization [40]. All twelve fimbrial operons were disrupted by multiple transposons in the TraDIS screen. No obvious host-specific phenotypes were seen, with a common pattern that mutants of fimbrial subunit genes were attenuated, whereas assembly genes were often dispensable, suggesting cross-talk in the assembly pathways. Stress responses are also important in the infection process, as *Salmonella* is subjected to a range of stresses including low pH, oxidative stress and heat shock [41]. The genetic components of these stress responses overlap [42], and many of these genes harboured transposons that resulted in attenuation. These included the sigma factor gene *rpoE* and its anti-sigma factor *resA*, the heat shock chaperone genes *dnaK* and *dnaJ* and the heat shock protease gene *degP* (*htrA*). Interestingly, several stress response genes are variably attenuated in the different hosts, suggesting species-specific stresses. These include the two-component regulatory system genes *envZ* and *ompR*, and the oxidative stress response genes *dps*, *katE* and *proV* which are all attenuated in pigs and cattle but show little or no attenuation in chickens. Conversely, transposon mutants in *clpB*, *clpP* and *clpX*, which encode proteases and are involved in the regulation of *rpoS*, are attenuated in chickens but not pigs or cattle.

Many *S.* Typhimurium genes beyond the classical virulence factors and stress response genes were revealed to be important for oral infection of livestock species. These include genes involved in nucleotide metabolism (*pyrCD*, *purADGH*, *dgt*, *dcd*, *guaA*, *pyrCD* and *carAB*), aromatic amino acid biosynthesis (*aroABCDE*), inorganic ion transport (*trkAH*, *znuABC*, *fepCDG*), protein synthesis (*tufAB*, *fusA*, *efp*, *rplI*, *rpsK*), protein export (*tatABC*, *yajC*) and many genes involved in carbohydrate metabolism. Additionally, numerous low GC clusters of genes with putative metabolic functions and multiple attenuating mutations were identified. Several global regulators, including *crp*, *smpB* and *dam*, result in attenuation in all three hosts, whereas another, *fnr*, appeared only to be important for infection in chickens. On occasion, TraDIS revealed functional data at a sub-genic level. For example, most of the insertions that disrupt the SPI-1 gene *sptP* result in attenuation, but insertions close to the 3′ end of the gene are tolerated. The gene *rpoC*, which encodes the β′ subunit of RNA polymerase, is essential in *S.* Typhimurium [43]. However, one transposon insertion in the chicken, pig and cattle dataset, and two in the mouse dataset, were identified close to the 3′ end of *rpoC*. These insertions would disrupt the extreme C-terminal end of the encoded protein, and were found to reduce the fitness of the mutants in the animal screens. Similarly, an insertion was found at the 3′ end of the essential *polA* gene which encodes DNA polymerase I, and this mutant was significantly attenuated in chickens, pigs and cattle.

The recent RNAseq-based analysis of the *S.* Typhimurium SL1344 transcriptome [44] identified a number of small-regulatory RNAs. As indicated in Table S4 several of these were implicated in colonization by TraDIS. For many it is difficult to demonstrate conclusively a colonization-associated phenotype from the TraDIS data alone, since we cannot preclude the potential for polar effects on adjacent genes. This is the case for InvR, which is encoded within SPI-1. Table S4 details only the sRNA genes annotated in the SL1344 genome (which differs from ST4/74 by just 8 SNPs [45]), but in the TraDIS data there are a number of attenuating transposons within large intergenic regions that could reveal the presence of novel sRNA genes.

### Putative host-specificity determinants of *S.* Typhimurium

The chicken, pig and cattle TraDIS data presented in Figure 4 indicate that a shared core set of 611 genes is required for efficient colonization of all three species, with a smaller set of species-specific colonization factors. The core set comprises approximately two thirds of the genetic requirements for infection of each individual species, and 48% of the total set of colonization-associated genes. There are 259 genes which are required for systemic infection of mice for which comparable data are available from the food-producing animals (Figure 4); of these, 140 also contribute to oral infection of chickens, pigs and cattle, and only 43 are putative mouse-specific factors. Many of the differences between the mouse and chicken/pig/cattle datasets may arise from the additional genetic requirements for infection via the oral route.

Although most colonization factors were necessary for infection of chickens, pigs and cattle, there were some patterns amongst the colonization factors that appeared to function in a host-specific manner that may reflect underlying differences in host biology. There are many genes associated with flagellar motility that are essential for infection of pigs but not required for chicken or calf infection, including *fliY*, *flgK*, *fliN*, *flgN*, *fliB* and *fliZ*. Several other flagellum-associated genes (*flgB*, *flgL*, *fliL*) are required for infection of cattle but not chickens or pigs. In chickens, many genes that are involved in anaerobic growth are required; these include genes involved in the production of group I hydrogenase (*hypOBF*, *hybABCDF*), fumarate reductase (*frdAD*), *pflB*, *pfkA*, rNTP reductase (*nrdDG*) and the global regulator Fnr. Differences in oxygen tension proximal to villus tips have been detected that modulate the regulation of *Shigella* virulence genes [46], therefore the requirement for distinct respiratory pathways by *S.* Typhimurium in food animals may reflect differences in the niches occupied. Also required in chickens, but apparently not calves or pigs, are the *virK* homologue *ybjX*, *ilvGE*, *clpB* and the *his* operon. The observation that many of the host-specific phenotypes were observed independently in multiple genes affecting the same pathways strongly suggests that these effects are due to differences in the within-host environment. For example, in relation to fumarate reductase there were nine independent *frdA* mutants that were all significantly attenuated in chickens; of these only one showed significant attenuation in pigs, and none in calves. Similarly, in relation to group I hydrogenase, from a total of 15 mutations affecting the *hybABCDF* genes, 12 showed significant attenuation in chickens, but none in pigs or calves (Table S2).

The serovar Typhimurium strain ST4/74 investigated here is a natural bovine isolate that elicits pathology typical of clinical salmonellosis in all the host species used in this study. However, some atypical *S.* Typhimurium strains exist that have lost the capability to colonize a broad range of hosts. For example, the laboratory-adapted strain LT2 and its derivatives tend to be avirulent or less virulent in mice relative to natural isolates of serovar Typhimurium [47] and ST4/74-based strains. It is noteworthy that the *rpoS* gene encoding the sigma factor σ^S^, which is defective in LT2 and associated with the relative avirulence of this strain [48], was found to be required in all four species tested by screening of the *S.* Typhimurium libraries we describe (Table S3). TraDIS identified additional regions of the ST4/74 genome that are required for infection, but which are absent from the LT2 genome. These include several genes that are encoded within the same phage element: SL1344_1965, which is required for infection of mice, and SL1344_1929/30 and SL1344_1976, which are required for infection of chickens, pigs and cattle. Moreover, some Typhimurium strains have become adapted to a particular host, and the genome sequence of a human-adapted variant [49] reveals the decay of a number of genes important in colonization of food animals (e.g. *allP*, *sseI*, *pipD*, *ydeE*), but also other pseudogenes for which no role in food animals for the intact gene could be detected (e.g. *ratB*, *ygbE*, *yhjU*). Integration of genome sequences with high-resolution functional data of the kind we describe will provide further clues to explain the differential virulence of host-adapted or laboratory strains relative to natural isolates.

Our study represents the first comprehensive genome-wide survey of the role of thousands of *Salmonella* genes during colonization of the primary reservoirs of human non-typhoidal salmonellosis. TraDIS simultaneously assigned the genotype and relative fitness of 7702 distinct *S.* Typhimurium insertion mutants in chickens, pigs and cattle, representing over 90% of the mutants screened in pools of up to 475 mutants per animal. TraDIS therefore represents a significant advance in the reduction, refinement and replacement of animal models relative to STM, where only negatively-selected mutants tend to be interrogated and the vast majority of insertion sites and phenotypes are unreported [50]. Multiple lines of evidence suggest that the TraDIS data are robust and reliably reflect the fitness of the screened mutants in each host animal. Many of the attenuated mutants were found to harbour transposons in genes known to be involved in colonization. The TraDIS fitness scores correlated well with established datasets obtained using STM [10], [12] and TMDH [21]. Multiple mutations within the same gene or pathway usually gave comparable phenotypes, and most of the attenuated mutants demonstrated the same phenotype independently in the three different food-producing animal hosts. The examples of putatively host-specific attenuation tended to be restricted to particular pathways with multiple independent mutations. Finally, analysis of defined knockout mutants of targets chosen based on the TraDIS data reproduced attenuated phenotypes in all but one case.

Many novel colonization-associated genes were identified within the *S.* Typhimurium genome and the data provide an invaluable resource for the community to mine and extend. Moreover, TraDIS indicated that thousands of mutations exerted little or no effect *in vivo*, implying functional redundancy that may limit and refine the selection of targets for novel inhibitors, as previously suggested [51]. Unlike library screens conducted in murine typhoid models to date, we provide highly relevant data for control of intestinal *S. enterica* infections in food-producing animals, and thus zoonosis. Attenuating mutations may be suitable for selection and refinement of live vaccines for food-animals, and these in turn may express heterologous antigens. Further, the data will guide the interpretation of existing and fast emerging datasets on the repertoire, sequence and expression of *Salmonella* genes and aid the modelling of virulence in a wider evolutionary and ecological context. Our data reflect mutant phenotypes at a specific time and site, and further studies on the temporal and spatial role of *Salmonella* genes are likely to be informative. This study also establishes TraDIS as a quantitative technology in functional genomics, which has potential for widespread application beyond the realm of microbial pathogenicity.

## Methods

### Ethics statement

Animal experiments were conducted according to the requirements of the Animals (Scientific Procedures) Act 1986 (project license number 30/2485) with the approval of the local Ethical Review Committee.

For full details of experimental animals, bacterial strains, materials, molecular biological techniques and statistical methods see Text S1. Briefly, a library of 8550 mini-Tn*5* mutants was generated in a spontaneous nalidixic acid resistant variant of *S.* Typhimurium ST4/74. The mutants were combined into pools of 95 for chickens, and 475 for pigs and calves. Animals were inoculated orally and killed humanely 4 days (chickens and calves) or 3 days (pigs) after infection, or earlier if the clinical endpoint was reached. A section of an appropriate tissue (whole caeca for chickens, spiral colonic mucosa for pigs and distal ileal mucosa for calves) was homogenized and grown overnight on MacConkey agar plates to isolate the output bacteria.

Genomic DNA was prepared from the inocula and output samples, and fragmented to ∼300 bp. An Illumina adapter was ligated to the fragments, and PCRs were performed using an adapter-specific primer in conjunction with primers homologous to each end of the transposon. The sequences of all oligonucleotide primers used in this study are detailed in Table S5. The resultant products were sequenced on single end Illumina flowcells using a sequencing primer designed to read a 10 bp tag of transposon-derived sequence, plus 27 bp of flanking genomic DNA. Sequences containing the tag were mapped to the *S.* Typhimurium SL1344 genome sequence. A transposon was inferred to be present if there were corresponding reads derived from each end of the transposon in the input pool.

The number of reads corresponding to each transposon in the input pool, and the number of reads mapping to the equivalent position in the output pool data, were compared using DESeq [52]. The ratio of input∶output read counts was determined, after normalisation to account for variations in the total number of reads obtained for each sample, and expressed as log_2_(fold change), referred to as the fitness score. A negative fitness score indicates an attenuated mutant, a positive score indicates a mutant which was more abundant in the output pool than in the input. For strongly attenuated mutants, no reads were obtained in the output pool, so it was not possible to calculate a finite log_2_(fold change); such mutants were assigned an arbitrary fitness score of −15. For each individual mutant, the hypothesis that the fitness score was equal to zero (*i.e.* that the mutant was present at equivalent levels in the input and output pools) was tested using the negative binomial distribution as implemented in DESeq. DESeq models variance under the assumption that mutants with comparable levels of sequence coverage exhibit similar levels of dispersion. We exploited this model to estimate *P* values for all mutants whilst minimising the number of biological replicates by fitting using only those mutants for which replicate data points were available, and applying the resultant model to the data derived from all mutants.

Defined null mutants were obtained for twelve genes identified as attenuated in the TraDIS screen, and assessed in competition with wild-type ST4/74 during oral infection of chickens. The ratios of mutant∶wild-type bacteria from caecal isolates at day 4, 6 and 10 were compared with those in the inoculum, and the significance of any differences was tested using Student's t-test.

## Supporting Information

Figure S1 Comparison of fitness scores obtained using TraDIS with the equivalent attenuation scores obtained using TMDH. Values were obtained by investigation of pools of *S.* Typhimurium SL1344 mutants screened during systemic infection of BALB/c mice using the two technologies.
(PDF)

Figure S2 Venn diagram showing the numbers of genes in which at least one significantly attenuated mutant was identified for each of the four host species.
(PDF)

Figure S3 Venn diagrams illustrating the overlap between attenuated and non-attenuated mutants from the earlier STM studies and the attenuation of mutants with transposon insertions at equivalent loci in the TraDIS datasets a) chickens, b) pigs and c) cattle [10], [12].
(PDF)

Figure S4 Illustration of the chorismate biosynthesis pathway, adapted from KEGG [24]. For each step, boxes indicate the EC numbers of the enzyme(s) mediating the specified reactions, and are coloured blue, if a mutant in the associated genes was attenuated during intestinal colonization of chickens and red if the gene was disrupted but not attenuated. White boxes indicate enzymes absent from *S.* Typhimurium SL1344. Mutants that are defective in multiple stages of the pathway are attenuated, however two of the steps can be catalysed by the products of multiple genes, so inactivation of the individual genes associated with these steps does not result in attenuation.
(PDF)

Figure S5 Box plot of GC content of genes for which attenuated mutants were observed in the chicken TraDIS dataset, and genes for which no attenuated mutants were obtained.
(PDF)

Table S1 Complete dataset derived from TraDIS investigation of pools of *S.* Typhimurium SL1344 mutants during systemic infection of BALB/c mice. The table includes the position in the genome and orientation of each transposon identified, identity of disrupted genes, the raw sequence counts, fitness scores, adjusted *P* values annotation of the predicted function of selected genes. ‘Absent’ indicates a gene that is present in SL1344 but has no identifiable orthologue in the LT2 genome. The equivalent TMDH attenuation scores are included for mutants that could be unambiguously identified in the TMDH dataset.
(XLSX)

Table S2 Complete dataset derived from TraDIS investigation of pools of *S.* Typhimurium ST4/74 mutants during intestinal colonization of chickens, pigs and cattle. The table includes the position in the genome and orientation of each transposon identified, and details of any disrupted gene. ‘Gene direction’ indicates the orientation of each gene (pink: sense, blue: antisense). Gaps in the fitness score columns indicate data points that were omitted due to stochastic loss of mutants in some animals, for example owing to recovery of output pools of inadequate size to be confident that mutants were absent owing to attenuation rather than chance.
(XLSX)

Table S3 List of all genes disrupted in the TraDIS mutant libraries from the food-producing animal and mouse experiments. For each gene, “yes” indicates that at least one significantly attenuated mutant was identified in that experiment, “no” indicates that the gene was disrupted but no evidence of attenuation was obtained.
(XLSX)

Table S4 Fitness scores of mutants within sRNA genes annotated in the *S.* Typhimurium SL1344 genome.
(XLSX)

Table S5 Oligonucleotides used in this study.
(XLSX)

Text S1 Supporting data and methods.
(PDF)
