# Supplementary figures and images for: Comprehensive Assignment of Roles for Salmonella Typhimurium Genes in Intestinal Colonization of Food-Producing Animals
Source: PLoS Genet. 2013 Apr 18;9(4):e1003456. doi: 10.1371/journal.pgen.1003456 (PMC3630085; doi:10.1371/journal.pgen.1003456)

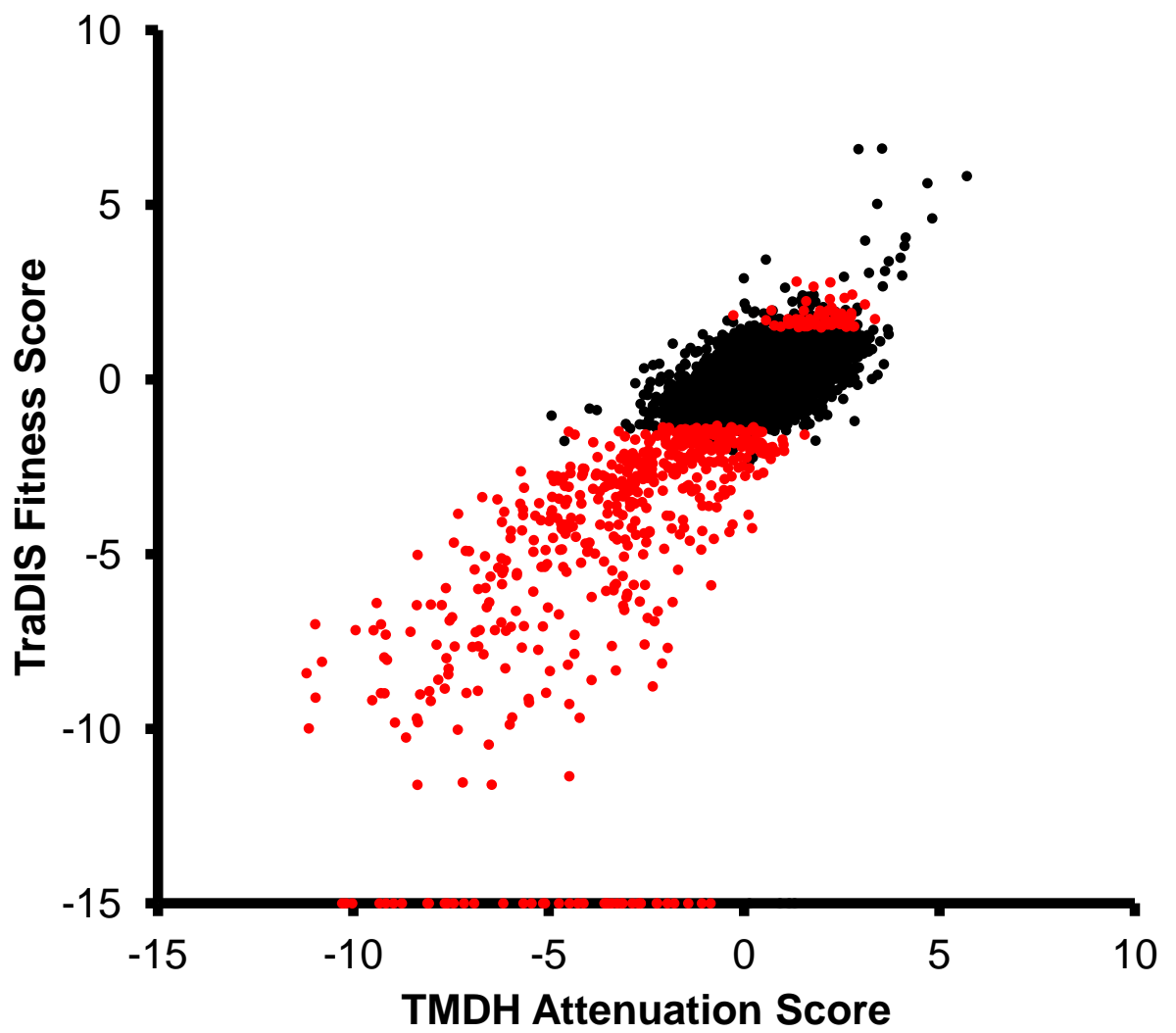

Supplement: Figure S1 — Comparison of fitness scores obtained using TraDIS with the equivalent attenuation scores obtained using TMDH. Values were obtained by investigation of pools of S. Typhimurium SL1344 mutants screened during systemic infection of BALB/c mice using the two technologies. (PDF) [file pgen.1003456.s001.pdf]

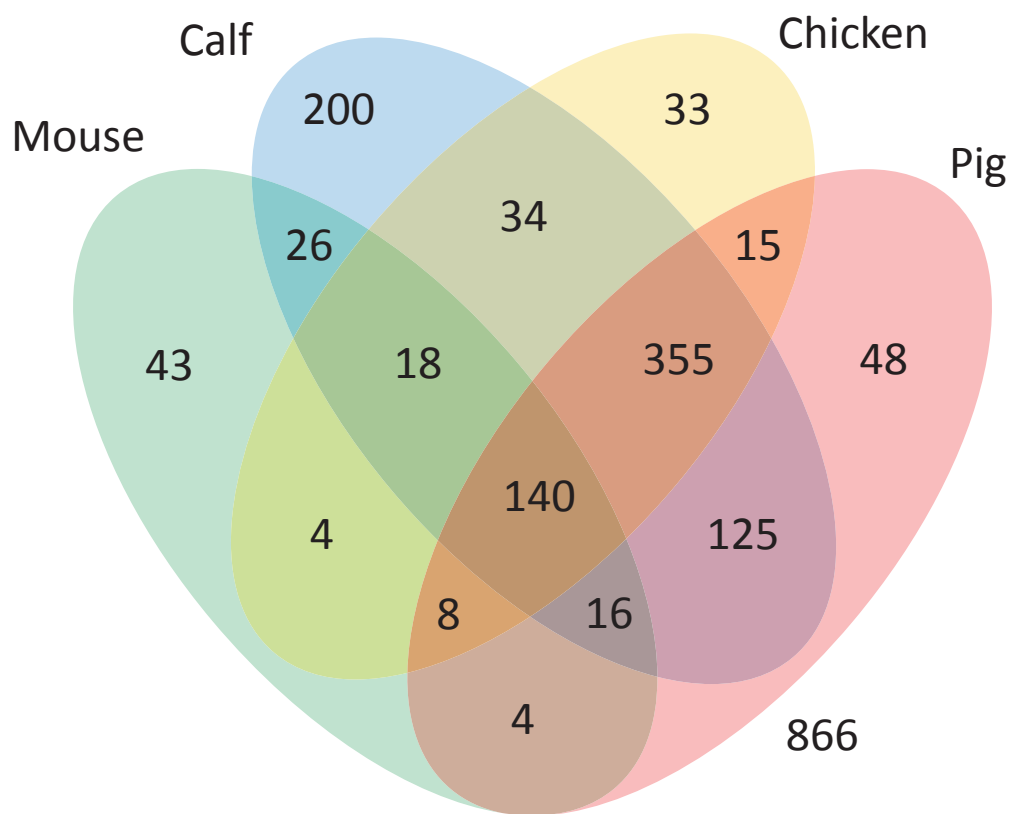

Supplement: Figure S2 — Venn diagram showing the numbers of genes in which at least one significantly attenuated mutant was identified for each of the four host species. (PDF) [file pgen.1003456.s002.pdf]

**a.** STM

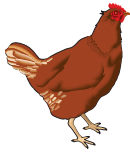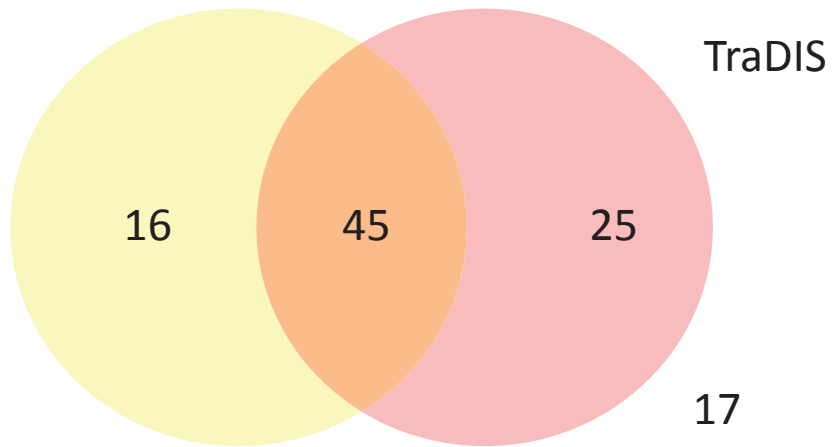

**b.** STM

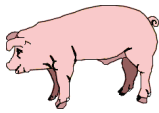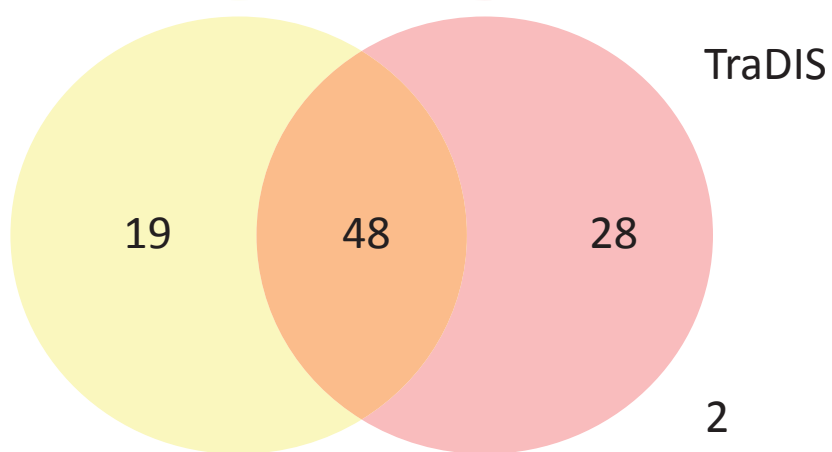

**c.** STM

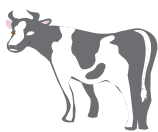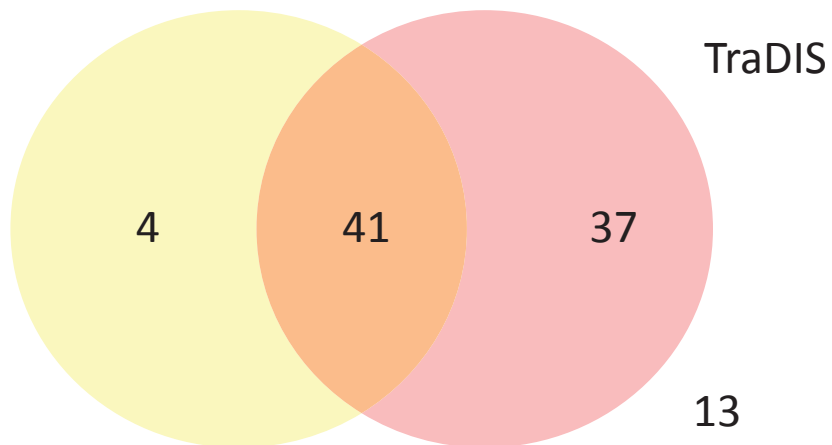

Supplement: Figure S3 — Venn diagrams illustrating the overlap between attenuated and non-attenuated mutants from the earlier STM studies and the attenuation of mutants with transposon insertions at equivalent loci in the TraDIS datasets a) chickens, b) pigs and c) cattle [10], [12]. (PDF) [file pgen.1003456.s003.pdf]

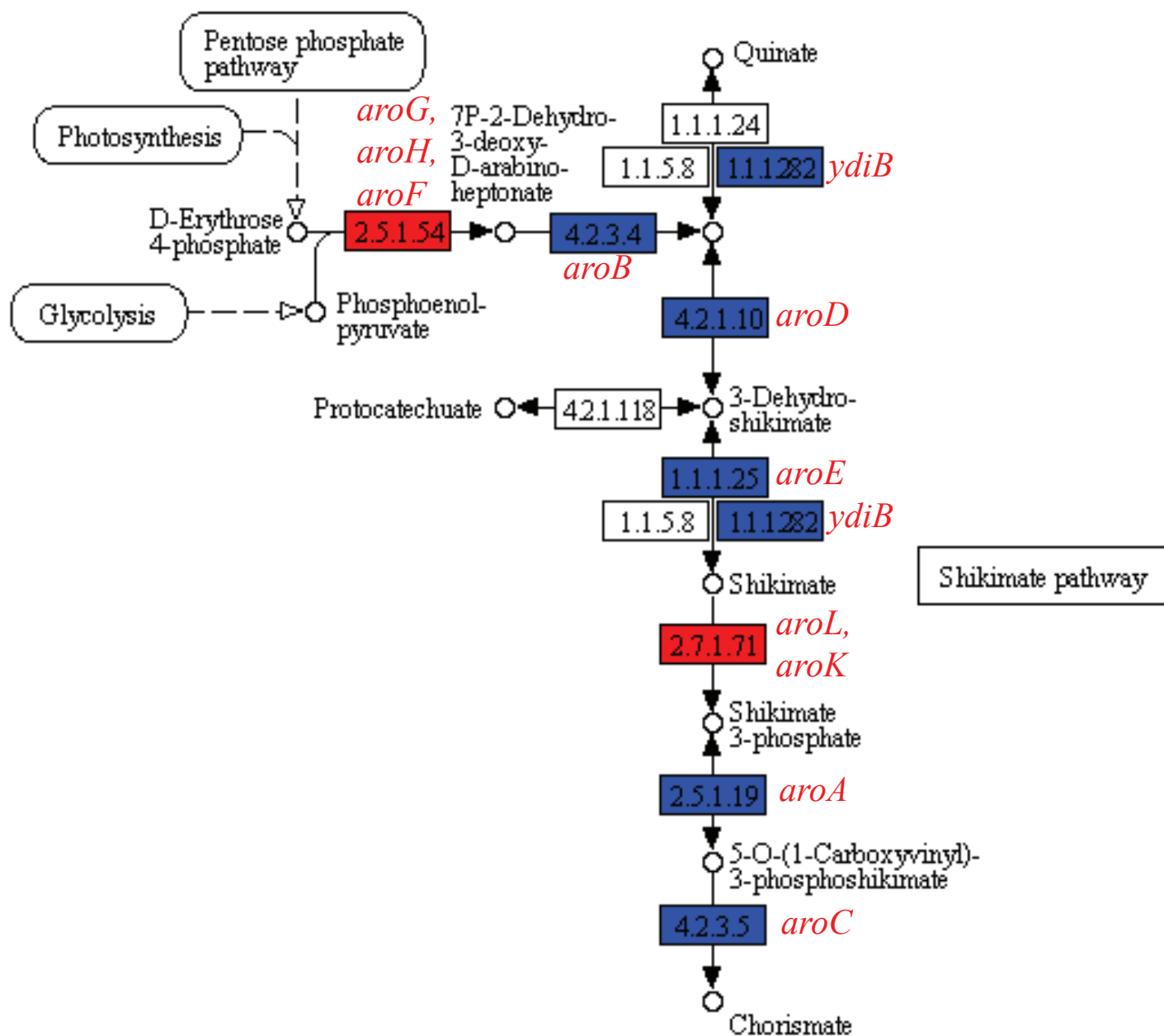

Supplement: Figure S4 — Illustration of the chorismate biosynthesis pathway, adapted from KEGG [24]. For each step, boxes indicate the EC numbers of the enzyme(s) mediating the specified reactions, and are coloured blue, if a mutant in the associated genes was attenuated during intestinal colonization of chickens and red if the gene was disrupted but not attenuated. White boxes indicate enzymes absent from S. Typhimurium SL1344. Mutants that are defective in multiple stages of the pathway are attenuated, however two of the steps can be catalysed by the products of multiple genes, so inactivation of the individual genes associated with these steps does not result in attenuation. (PDF) [file pgen.1003456.s004.pdf]

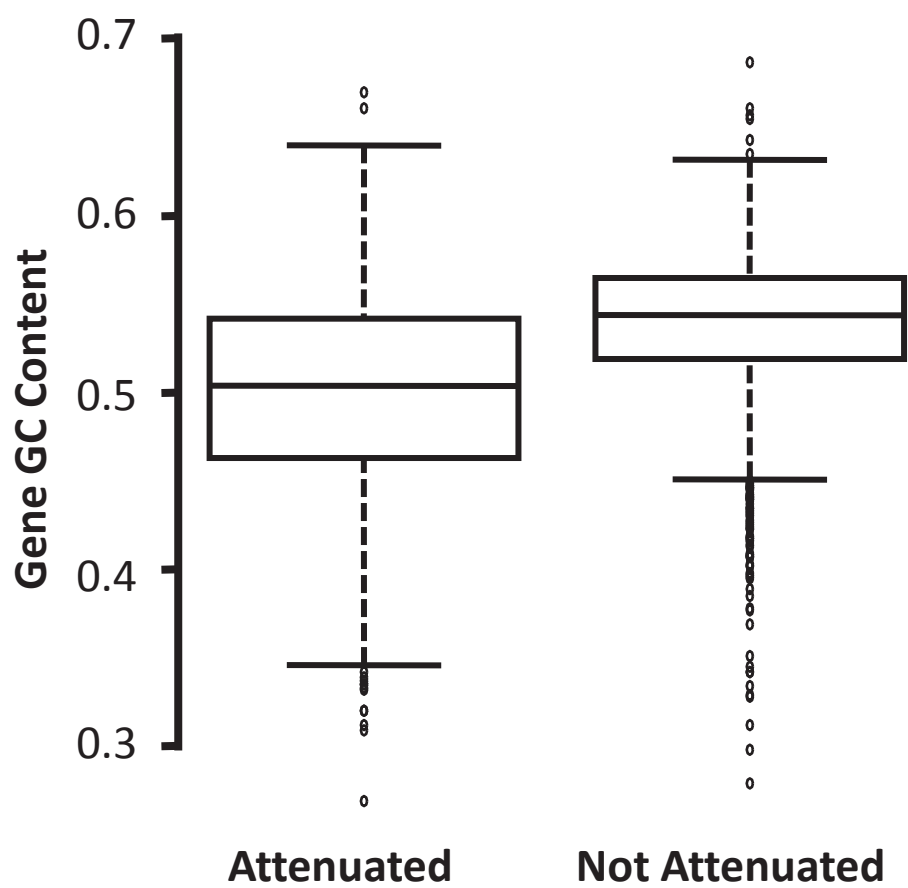

Supplement: Figure S5 — Box plot of GC content of genes for which attenuated mutants were observed in the chicken TraDIS dataset, and genes for which no attenuated mutants were obtained. (PDF) [file pgen.1003456.s005.pdf]
