# Supplementary material for: Comprehensive Assignment of Roles for Salmonella Typhimurium Genes in Intestinal Colonization of Food-Producing Animals
Source: PLoS Genet. 2013 Apr 18;9(4):e1003456. doi: 10.1371/journal.pgen.1003456 (PMC3630085; doi:10.1371/journal.pgen.1003456)
Supplement: Text S1 — Supporting data and methods. (PDF) [file pgen.1003456.s011.pdf]

Supplementary Data

Comparison of data obtained for SL1344 mutants screened in mice by TraDIS or microarray-based TMDH

To test whether TraDIS provides reliable quantitative datasets, the technology was applied to investigate a pool of 5184 Mu and 5184 mini-Tn*5* transposon mutants screened during systemic infection of BALB/c mice [4]. The TraDIS fitness scores were compared with the attenuation scores obtained by analyzing the same mutant pools using the microarray-based TMDH method [4]. In total, 9792 transposon insertions (4992 Mu and 4800 Tn*5*) were unambiguously mapped to the SL1344 genome by TraDIS (94.4% of mutants screened). In the TMDH analysis, the locations of 8533 transposons were identified (82.3%), indicating the improved resolution of TraDIS. Based on transposon identity (Mu or Tn*5*), insertion location and orientation, 7087 mutants from the TraDIS dataset had equivalents that could be identified unambiguously in our earlier TMDH analysis [4] (see Table S1). The remainder includes 1857 mutants identified by TraDIS which could not be identified in the TMDH dataset. For a further 848 mutants, the TraDIS dataset was compatible with two or more mutants in the TMDH dataset, so it was not possible to determine the corresponding mutant unambiguously. This reflects the inherent limitations of TMDH, since transposons can only be localised to a range of bases between two adjacent microarray probes, some microarray signals may reflect multiple transposon insertions, and transposons can be missed if they are inserted into a region of the genome not covered by the microarray, or within a small restriction fragment.

Table S1 indicates that a number of mutants scored as non-attenuated by TMDH are identified as attenuated by TraDIS. This reflects one of the key advantages of TraDIS over its predecessor. In TMDH and similar methods, multiple mutants are assessed on the same

microarray, and the signals from transposons that are in similar genomic locations can be confounded. This can lead to an attenuated mutant being missed if there is a non-attenuated mutant close by, for example in the adjacent intergenic region. Although the signal from the attenuated mutant is absent in the output pool, the effect is masked by the signal from the non-attenuated mutant.

Examples of mutants identified as attenuated in mice by TraDIS that had been missed by TMDH include *secA*, which encodes part of the general secretory pathway and has a well-established role in virulence [5]; *fis*, which encodes a DNA-binding protein that is involved in transcriptional regulation of genes associated with virulence [6]; the lipopolysaccharide biosynthesis genes *waaD* (*rfaD*) and *wbaU* (*rfbU*); the ATP synthase gene *atpD*; *rpoZ,* which encodes the ω-subunit of RNA polymerase; the ferredoxin gene *fdx*; and the putative outer membrane lipoprotein gene *ytfJ*.

Assessment of stochastic loss of mutants in food-producing animal TraDIS

Using complex pools of mutants in competitive infection experiments may result in problems with spontaneous ‘drop-out’ of some mutants due to a population bottleneck during the infection. Data obtained from animals exhibiting drop-out is not biologically meaningful since mutants may be negatively-selected in output pools for reasons unrelated to their genotype. Pilot studies indicated that pools of 475 mutants could be screened in pigs and calves and 95 mutants screened in chickens with little evidence of stochastic loss and reliable negative-selection of the same mutants across replicate animals given the same pool (data not shown). At higher pool complexities (950 mutants in calves or pigs and 475 mutants in chickens) negative selection of specific mutants was not reliably observed, meaning that mutants may appear absent for reasons unrelated to their genotype.

For the samples obtained from food-producing animals, it was desirable to be able to distinguish the TraDIS data derived from each individual animal. This was accomplished

using multiplex sequencing (see Supplementary Methods) and is necessary as outbred conventionally-reared farm animals show more inter-animal variation than inbred mice, and some animals may be more prone to random drop-out. Had data from multiple animals been pooled without such multiplex sequencing, it would not be possible to specify which mutants were screened in which animal, or whether absence of specific mutants was a consequence of a high level of stochastic loss in a single animal or recovery of a pool of an inadequate size. Output pools from each animal were assessed for the possibility of drop-out prior to TraDIS analysis by a preliminary TMDH analysis (data not shown) and assessment of the number of bacteria recovered. Any experiment showing evidence of stochastic loss of mutants was repeated. Analysis of the TraDIS data revealed evidence of random drop-out in one chick, two calf and two pig experiments, manifested as a skewed distribution of fitness scores with a high proportion of mutants appearing to be attenuated. Data derived from these animals were omitted from subsequent analysis.

Comparison of fitness scores assigned to *S.* Typhimurium mutants in food-producing animals by TraDIS with STM findings

A previous study surveyed 1,045 *S.* Typhimurium 4/74 mutants for their ability to colonize the same intestinal sites of chickens, pigs and calves and identified 227 attenuating mutations [2,3]. Although different mutant libraries were used, the strain background and infection protocols were identical, so a comparison of the two datasets is of interest. From the STM study, sequence data were available for 191 mutants. Of these, there were 111 that had a corresponding mutant in the TraDIS library for which the transposon disrupted the same gene/intergenic region (although rarely at precisely the same position). Unusually for an STM study, the available data include some non-attenuated mutants for each host, since all mutants that were attenuated in any of the hosts were characterized by sequencing. The Venn diagrams in Figure S5 show the overlap between genes/intergenic regions associated with

attenuated mutants in the STM study, and the attenuation of equivalent mutants in the TraDIS data. For the calf and pig datasets, the association between the STM and TraDIS predictions of attenuation is significantly non-random (Fisher's exact test, calf: *P*=0.035; pig: *P*=0.017). However, this was not the case for the chicken dataset (*P*=0.139), largely due to the impact of genes from SPI-1, SPI-2 and SPI-4, which were not identified as essential for virulence in chickens in the STM study. Mutants within these pathogenicity islands showed impaired fitness in chickens in the TraDIS experiments, although for most mutations the extent of attenuation was less pronounced than in calves.

The density of insertions and the ability to derive per-gene fitness scores raised the issue of the sensitivity of the two methods. For example, we previously indicated that SPI-4 plays a host-specific role, being required for intestinal colonization of calves, but not chickens[17] or pigs[18]. SPI-4 encodes a Type I secretion system that secretes SiiE [7,8]. Almost all (60/61) of the mutants in *siiE* are significantly attenuated in calves. However, many of the *siiE* mutants are also attenuated in chickens (33/68) and pigs (36/57). Closer inspection reveals that transposons within the central repetitive region are tolerated in chicks and pigs, but result in attenuation in calves. Insertions elsewhere in the gene result in attenuation in all three hosts. Thus, whilst it may be correct to infer that the magnitude of the phenotype of *siiE* mutants is greatest in calves, the gene nevertheless contributes to colonization of other hosts. The reliable negative-selection of *siiE* mutants and those lacking other secreted factors also indicates that complementation of mutant phenotypes *in trans* by other pool members may not be a significant issue at the pool complexities used.

Although the pig and cattle STM datasets are in broad agreement with the equivalent TraDIS data, there are cases of discrepancies for all three hosts. This may simply reflect the use of different transposon libraries for the two studies, since the genes will not be disrupted in the same position and hence may not exhibit a comparable phenotype. However, it should

also be noted that STM relies upon highly subjective visual assessment of the intensity of hybridization signals between input and output pools and between co-screened mutants. It is therefore likely to miss many attenuating mutants revealed by the more sensitive and quantitative TraDIS analysis. Recently, we retrospectively applied TraDIS to a library of *E. coli* O157:H7 mutants that had previously been investigated by STM [9]. In that study, STM was insufficiently sensitive to detect attenuated mutants present in the library with defects in genes with a well-established role in colonization of cattle, including the locus of enterocyte effacement (LEE) genes encoding intimin and Tir as well as tens of Type III secreted effectors. This suggests that TraDIS should be considered the gold standard technology for transposon mutant screens, and that discrepancies with the existing datasets are likely due to the inherent limitations of STM.

Association of attenuated mutants with GC content

During the analysis of the TraDIS data it became clear that many regions of the genome with low GC content are important for intestinal colonization of chickens, pigs and cattle (see Figure S4). Low GC tracts of the genome often represent regions that have been acquired relatively recently by horizontal transfer, and do not yet exhibit the native GC content of the *S*. Typhimurium genome [10]. The low GC regions that are important for infection include established pathogenicity islands such as SPI-1 and SPI-2, but also many metabolism-associated islands. The H-NS transcriptional repressor preferentially binds to the curved DNA that is characteristic of low GC regions of the genome, and is thought to play a role in gene silencing of horizontally acquired DNA [11,12]. The involvement of low GC regions in colonization of chickens, pigs and cattle suggests that many genes have undergone the required adaptations to circumvent this silencing [13].

Intragenic variation in fitness scores

For many genes, several transposon insertions were identified at distinct locations in the coding sequence. In some cases, *e.g. slrP*, considerable variation is seen in the fitness scores observed for different insertions within the same gene. For mutants that were screened in different pools, this could reflect distinct competition dynamics, or variation in the immune responses between the individual host animals. Alternatively, insertions at specific positions may produce varied fitness scores depending on their impact on the activity of the encoded product; for example, it is more likely that an insertion close to the stop codon will be better tolerated than an insertion in the central portion of the gene. This can be considered an advantage of TraDIS over studies with defined deletion mutants, since position-sensitive phenotypes provide information at sub-genic resolution, as we describe for *rpoC* (RNA polymerase β' subunit) and *polA* (DNA polymerase). If conducted at adequate saturation, TraDIS can therefore be considered comparable to a truncation assay, in which the minimal portion of the gene that is required for function is delimited. However, it is also possible that production of a truncated protein may produce an aberrant phenotype, as was the case in early studies of *pagC* mutants [14], where a transposon disrupting the signal sequence was found to result in a truncated protein product that was incorrectly localized to the inner membrane and resulted in an attenuated phenotype that did not reflect the behaviour of a null mutant. Although such phenomena are unlikely to be widespread, it is important to be aware of the genomic context of each transposon insertion when interpreting its associated phenotype. Similar consideration must be made of the possibility of transposon insertions having polar effects on genes downstream of the site of insertion. We encourage use of our online resource (http://www-tradis.vet.cam.ac.uk) to interpret the TraDIS data in the context of transcriptional and genetic organization of the disrupted loci.

Supplementary Methods

Bacterial strains and plasmids

The library of 10,368 mutants of *S.* Typhimurium strain SL1344 harbouring insertions of Tn*5* and Mu transposons modified to incorporate outward-facing T7 and SP6 promoters has been described [4]. SL1344 is a *hisG* auxotroph derived by transduction from the calf virulent strain ST4/74 [15], previously known as S2337/65 [16,17]. Though highly virulent in inbred mice, pilot studies with the SL1344 mutant library indicated that it failed to colonize the intestines of chickens, pigs and calves at the levels previously described for a spontaneous nalidixic acid resistant derivative of ST4/74 [2, 3] and data not shown]. A separate mutant library was therefore generated in ST4/74 nal^R. Strains were cultured in Luria-Bertani (LB) medium or on LB, MacConkey or brilliant green agar supplemented with appropriate antibiotics at 37°C. Antibiotics were used at the following concentrations: nalidixic acid (Nal; 20 μg ml^{-1}), kanamycin (Kan; 50 μg ml^{-1}), ampicillin (Amp, 100 μg ml^{-1}). Plasmids pKD4 and pKD46 [18] were obtained from the *E. coli* Genetic Stock Centre, Yale University. Bacterial strains were stored in LB medium containing 15% (v/v) glycerol at -70°C.

Generation of a library of S. Typhimurium ST4/74 Nal^R transposon mutants

A derivative of EZ-Tn*5* <R6Kγori/KAN-2> (Epicentre Biotechnologies) was used to mutate ST4/74 Nal^R essentially as described [19]. The transposon possesses outward facing T7 and SP6 promoters but lacks the *ori*R6Kγ origin and was amplified from plasmid pCR2.1-TOPO-Tn*5* using a phosphorylated oligonucleotide 5’ CTGTCTCTTATACACATCTC 3’ with PfuUltra II Fusion HS DNA polymerase (Stratagene). Plasmid template DNA was removed by restriction digestion with *Dpn*I. Transposome complexes were created by mixing 100ng of isolated transposon in 1μl with 2μl EZ-Tn*5* transposase (Epicentre Biotechnologies) and 1μl 100% glycerol, incubated at ambient temperature for 3.5 h and used immediately thereafter. Bacterial cells for transformation were grown in LB broth to an optical density at

600nm (OD_{600}) of 0.5-0.8, harvested, washed three-times in 10% (v/v) glycerol and resuspended in 1/100 starting volume in 10% (v/v) glycerol. Cells were electroporated with 1µl of the transposome complex using a Bio-Rad Gene Pulser II in a cuvette with a 1 mm electrode gap at 2.2kV, 25µF and 200Ω. Transformants were recovered in 1 ml SOC medium at 37°C for 1h with aeration, spread on tryptic soy agar supplemented with 20µg/ml nalidixic acid and 50µg/ml kanamycin and incubated at 37°C overnight. Colonies were picked and grown overnight at 37°C in LB broth supplemented with Nal and Kan in microtitre trays and stored in 15% (v/v) glycerol in LB at -70°C. The library of 8550 mutants was generated from two separate transformations with different transposome complexes derived from the same transposon preparation. It is evident from analysis of the distribution of insertions on circular diagrams of the ST4/74 chromosome and two plasmids that a near random distribution was obtained (Figure 2).

Experimental animals

Animal experiments were conducted according to the requirements of the Animals (Scientific Procedures) Act 1986 (project license number 30/2485) with the approval of the local Ethical Review Committee. 25-32-day-old Friesian crossbred bull calves from commercial farms were used as described [20]. 42-day-old Large White × Landrace or White Durock × Landrace pigs were obtained from commercial farms and were used as described [3]. Calves and pigs were confirmed to be culture-negative for *Salmonella* by enrichment of rectal swabs overnight in Rappaport and selenite broth followed by subsequent culture on modified green agar supplemented with 120 µg ml^{-1} sulfadiazine. Unsexed, specific-pathogen-free (SPF) Light Sussex chicks from the Institute for Animal Health flock were reared together with access to dried food pellets and water *ad libitum*. Chicks were infected orally at 14-15 days old.

Screening of transposon mutant libraries

The SL1344 mutant library was screened in 20 pools of 480 mutants and 2 pools of 384 mutants by intravenous inoculation of inbred mice as described [4]. Approximately 10^6 colony-forming units (CFU) were delivered in 0.2ml PBS via the tail veins of duplicate six- to eight-week-old BALB/c mice (Harlan). For inoculation of food-producing animals, ST4/74 nal^R transposon mutants were replicated into 96-well microtitre trays containing 150 µl of LB broth supplemented with Nal and Km and grown statically overnight at 37°C. Mutants were combined randomly into pools of 95 for inoculation of chickens, and 475 for inoculation of calves and pigs. A total of 90 chickens, and 18 pigs and calves were required to screen all the mutant pools. 90 additional chickens were used to perform a complete biological replicate experiment, and partial replicates were performed for the other hosts using an additional 6 pigs (used to investigate three pools in triplicate) and 2 calves (to investigate two pools in duplicate). Data on the reliable performance of the same pool in repeated animals are available from the authors on request. 40 ml of each pooled culture was used for preparation of 'input pool' genomic DNA (see below).

For each pool, calves were fasted for 18 h prior to infection and orally dosed with 8.3×10^7 - 2×10^8 CFU in reconstituted powdered milk then were killed humanely 4 days after infection. A single calf had to be killed at day 3 post-inoculation as a humane end-point was reached. A section of distal ileal mucosa was aseptically excised at post mortem examination and 1 g full thickness biopsies were homogenized in 9 ml 0.9% (v/v) saline. A 200 µl aliquot was plated for viable counts on MacConkey agar supplemented with Nal and Km, and the remainder was plated in 1ml aliquots on 10 MacConkey agar plates supplemented with Nal and Km and incubated at 37°C overnight. Mean recoveries were 3.31×10^6 CFU/g of tissue.

Pigs were fasted for 18 h prior to infection and dosed orally with $1.3\text{-}2.9\times10^{10}$ CFU and killed humanely at 3 days post-inoculation (3 pigs were killed on day 2 after infection). A

section of mucosa from the spiral colon was excised at post mortem examination and homogenized and plated as for calves. Mean recoveries were 1.54×10^7 CFU/g tissue.

Pairs of chickens were dosed orally with $2.45\text{-}6.15 \times 10^8$ CFU and killed humanely 4 days post-infection. Both caeca (including contents) from each bird were removed, weighed and homogenized in 9 volumes of 0.9% (v/v) saline and 10 × 1 ml aliquots were plated as for calves. Mean recoveries were 2.9×10^8 CFU/g. Bacteria or output pool DNA recovered from independent caeca and duplicate birds were combined for a given pool of 95 mutants. In all cases, the population sizes sampled (relative to the complexity of the screened pool) are adequate to state at the ≥ 95% confidence interval that mutants are absent owing to attenuation as opposed to by chance, as inferred from a model for sampling populations for characteristics [21]. The anatomical sites sampled are key sites of persistence of *S.* Typhimurium in the respective hosts and enabled us to balance the requirement to recover output pools of an adequate size against the need to leave an adequate time for negative or positive selection to be evident.

Preparation of genomic DNA

For the input pools, bacteria were collected from 40 ml of pooled overnight culture by centrifugation at 10,500 *g* for 20 min at 4°C. For the 'output pools', bacteria from each set of 10 confluent plates were harvested into a total of 20 ml LB broth and cells from a 1.5 ml aliquot were harvested at 13,000 *g* for 2 minutes at ambient temperature. In each case the pellet was resuspended in 5 ml of TE buffer (10 mM Tris HCl, 10 mM EDTA, pH 8.0), then 20 µl of 50 mg ml^{-1} lysozyme (Sigma) were added and incubated at 42 °C for 1-2 h. 50 µl of proteinase K (Qiagen), 10 µl of RNase A (Qiagen) and 0.5 ml of 10% (w/v) N-lauryl sarcosine (Sigma) were added and incubated at 56°C overnight. Lysates were extracted by adding 1 volume of Ultrapure Phenol (Sigma) and centrifuged at 3,300 *g* for 20 min. Phenol-

extracted lysate was further purified using a Qiagen DNeasy blood and tissue DNA extraction kit, and the DNA was resuspended in 50 µl TE buffer.

TraDIS procedure

Optimization of the TraDIS methodology has been described [19]. For analysis of the library screened in mice, genomic DNA was fragmented to an average size of 300 bp by Covaris AFA [22], and an Illumina adapter ligated according to the manufacturer's instructions, with a modification that 1.5× the recommended reagent volumes were used in each step. Following ligation, fragments were separated in a 12 cm 2% agarose gel in 1× TBE buffer, at 6 V cm^{-1} without the preceding column clean-up step. Following electrophoresis for 45 minutes, 250-350bp fragments were excised, and DNA was extracted from the gel slice without heating [22]. The DNA was quantified using qPCR [22]. 22 cycles of PCR were then performed on 100ng of template genomic DNA using an adapter-specific primer (Sigma, HPLC purified) and a primer specific to one end of the transposon. Primer sequences are listed in Table S5. Amplified libraries were purified using a QiaQuick PCR purification column following the manufacturer's instructions, eluted in 30 µl EB, and then quantified by qPCR [22].

A minor modification of the above procedure was implemented for TraDIS analysis of the library screened in chickens, pigs and cattle. For the mouse studies, size-selection of the ligated Illumina fragment library was performed prior to PCR amplification. Since the transposon can be located anywhere within the fragment, size selection at this stage of the procedure does not guarantee a large amount of flanking genomic DNA. If the transposon is located at the end of the fragment, close to the adapter, then the resulting sequence will be mostly derived from the adapter and hence be difficult to map. This situation was avoided for TraDIS analysis of the library screened in food-producing animals, with size selection delayed until after the PCR step, ensuring that all the fragments contained sufficient genomic

DNA, and increasing the proportion of sequence reads that can be mapped. With this exception, the TraDIS procedure for analysis of inocula and output pools screened in chickens, pigs and cattle was identical to the mouse experiments.

The DNA fragment libraries were sequenced on single end Illumina flowcells using an Illumina GAII sequencer, for 36 or 54 cycles of sequencing, using a custom sequencing primer (see Table S5) and 2×hybridization buffer (10× SSC + 0.2% Tween-20). The sequencing primers were designed such that the reads consisted of 8-10bp of transposon-derived sequence, with the remainder derived from the region of the genome immediately adjacent to the transposon.

Multiplex TraDIS

For the mouse TraDIS experiments, samples using the same transposon were pooled, giving six samples in total, corresponding to the input and two replicate output pools for the Mu and mini-Tn*5* transposons. Each of these samples was sequenced on two Illumina GAII lanes, corresponding to the 5' and 3' ends of the transposon.

For the samples from food-producing animals , a strategy was devised to minimize the required number of sequencing lanes whilst retaining the ability to distinguish samples derived from each individual experimental animal. To enable this we exploited multiplex sequencing using error-corrected bar-coded primers [23]. Samples from each individual animal could have been individually bar-coded, but this level of complexity was unnecessary as the grouping of mutants was preserved across species. Input and output samples from each individual calf were bar-coded separately, and this information was also used to distinguish the pig samples, since the pools of mutants were identical. For the chick samples, smaller pools of mutants were used (95 mutants), with the total set of mutants from five chicks corresponding to those given to a single calf and pig (475 mutants). The chick samples were therefore labelled with five different bar-codes (one for each pool of 95 mutants, using pools

obtained from pooled caeca of duplicate chicks), and the chick and calf bar-codes were used in conjunction to identify data derived from each individual chick. For each host species, the input and output pools (including the available replicates) were pooled, and distinguished using additional multiplex tags. A total of six Illumina lanes were used, one each for the 5' and 3' end of the transposon for the three host species.

TraDIS sequence analysis

The transposon tag sequences were removed from the sequence reads using a custom Perl script available from the authors on request. Only sequences that had a perfect match to the Tn*5* or Mu tag sequence were included in downstream analyses. Following removal of the tag, the remainder of each sequence was mapped to the *S.* Typhimurium SL1344 genome sequence [24] (Genbank accession numbers FQ312003, HE654724, HE654725 and HE654726) using NovoAlign (Novocraft Technologies Sdn Bhd. http://www.novocraft.com). The SL1344 chromosome and plasmid sequences are identical to those of ST4/74 with the exception of 8 single nucleotide polymorphisms, one of which was in *hisG* [25]. All subsequent analyses were performed using R version 2.8.0 [26]. To filter out artefacts derived from incorrectly mapped reads or chimaeric sequences, we adopted the strict criterion that the presence of a transposon would be inferred only if corresponding sequences from both ends of the transposon were found in the input pool.

To quantify any changes in the number of reads arising from a specific insertion, between the input and output samples, we used the R package DESeq [27], software initially intended for the analysis of RNAseq count data but equally applicable to TraDIS. Because of the smaller number of inserts compared to the *in vitro* TraDIS analysis [19], we initially performed the analysis on a per-insert site, rather than per-gene, basis. The number of reads found at each insertion location was used as a separate feature count for input into DESeq. The data from each end of the transposon were summed, effectively treating the two datasets

as technical replicates. Log_2-fold change values were calculated to represent the difference in abundance of each mutant in the output pools relative to the input, and these were used as a measure of fitness equivalent to the TMDH attenuation scores. *P* values were estimated using DESeq, based on the correspondence between the available biological replicates, and adjusted for multiple testing using the Benjamini and Hochberg false discovery rate [28]. In contrast to typical RNAseq experiments, biological replicates were only available for a subset of mutants in the pig and calf experiments. To allow estimation of *P* values for all mutants, those mutants with available replicates were analysed initially using DESeq, and the dispersion curve obtained from these data was used to predicted the dispersion for mutants which lacked biological replicates.

Insertion of Tn*5* or Mu transposons results in a duplication of 9bp or 5bp, respectively. This means that the position of the transposon as determined by sequencing from either end differs by the length of the duplication. To simplify subsequent analysis, the position of the transposon was defined as being immediately 5’ of the central base in the duplicated region. For each transposon, any SL1344 genes that were disrupted were identified.

For summary purposes it is desirable to have a single fitness score associated with each mutated gene. To accomplish this, a ‘per-gene’ analysis was also performed by summing the read counts for all transposon mutants within each gene. These gene count data were analysed using DESeq in the same way as the ‘per-mutant’ counts, to obtain a fitness score and *P* value for each gene. These summary values should be interpreted with caution, as they do not take into account variation in the fitness of mutants bearing transposons at different positions within the gene, or differences in competition dynamics between mutants with defects in the same gene but screened in distinct pools. This only affects a small proportion of the dataset, and a per-gene fitness score allows a number of additional analyses to be performed.

Construction of defined mutants

S. Typhimurium genes *carB*, *clpB*, *ilvC*, *virK*, *mig-14*, *ytfL*, SL1344_0084 (STM0084), *pagN*, and SL1344_4248 (STM4312) were mutated by λRed recombinase-mediated integration of linear PCR products. Primers were designed to amplify the pKD4-encoded Kan resistance cassette, including approximately 40-bp extensions from the 5' and 3' ends of the genes to be mutated (Table S5). PCR products were purified and electroporated into ST4/74 (pKD46) induced to express λRed recombinase as described [14]. Kan^R recombinants were selected and cured of pKD46 by culture at 37°C in the absence of ampicillin. Mutations were confirmed by PCR with primers specific to the Kan cassette (k1 and k2) and primers flanking the target genes (Table S5).

Insertions in SL1344_3128 (STM3154) and *trxA* were transferred to ST4/74 nal^R by transduction with bacteriophage P22/HT*int*. Briefly, donor lysates of P22 phage were prepared by the addition of P22 phage to a broth culture of *S.* Typhimurium 14028 ΔSTM3154::Kan [29] or SL1344 *trxA*::Kan^R [30]. Cultures were reincubated at 37°C for 18 h without aeration. Bacteria were removed by centrifugation at 5,000 *g* 10 min, 4°C and the supernatant filtered through a 0.45μm pore size membrane filter. The resulting lysates were separately added to a broth culture of recipient *S.* Typhimurium ST4/74 Nal^R. After static incubation at 37°C for 20 min, the mixture was plated onto LB agar containing Nal and Kan. Following overnight incubation at 37°C, resulting transductants were re-streaked onto green indicator agar [31] to check for the presence of pseudolysogens. Mutations in non-pseudolysogenic transductants were confirmed by PCR with primers flanking the target gene (Table S5).

A *zirT*::Kan^R mutant was generated by allelic exchange using pOG-RzirT-Km [32] (a kind gift from Brett Finlay, University of British Columbia) as previously described [8]. The presence of the mutation was confirmed by PCR using primers flanking *zirT* (Table S5). All

mutants were tested for non-agglutination with 5% (w/v) acriflavine-HCl to confirm normal LPS synthesis.

In vivo co-infection study

Mixed infections with mutant and wild-type strains were used to assess the phenotype of selected candidate virulence genes identified by TraDIS. Cultures of wild-type ST4/74 Nal^R and mutant strains marked by inserted kan^R cassettes were mixed in a 1:1 ratio prior to oral inoculation of 15-day-old Light Sussex chicks with 2.27-4.60×10^8 CFU. A group of birds was separately inoculated with ST4/74 Nal^R wild-type only, at 3.45×10^8 CFU, as a control. Three birds per group were removed for post mortem examination at 4, 6 and 10 days post-inoculation. Both caeca (including contents) from each bird were removed, weighed and homogenized in 9 volumes 0.9% (v/v) saline. A 200µl aliquot was diluted and plated for viable counts on MacConkey agar supplemented with Nal to enumerate total numbers of bacteria and MacConkey agar supplemented with Nal and Km to enumerate mutant bacteria. The number of viable wild-type bacteria was calculated by subtracting the count on medium containing Nal plus Km from that on medium containing Nal alone. Competitive indices (CIs) were calculated as the ratio of mutant to wild-type in the output pool divided by the ratio of mutant to wild-type in the inoculum. Data are presented as the mean CIs ± standard errors of the means.

Supplementary References

1. Kanehisa M, Goto S, Hattori M, Aoki-Kinoshita KF, Itoh M, et al. (2006) From genomics to chemical genomics: new developments in KEGG. Nucleic Acids Res 34: D354-357.

2. Morgan E, Campbell JD, Rowe SC, Bispham J, Stevens MP, et al. (2004) Identification of host-specific colonization factors of *Salmonella enterica* serovar Typhimurium. Mol Microbiol 54: 994-1010.

3. Carnell SC, Bowen A, Morgan E, Maskell DJ, Wallis TS, et al. (2007) Role in virulence and protective efficacy in pigs of *Salmonella enterica* serovar Typhimurium secreted components identified by signature-tagged mutagenesis. Microbiology 153: 1940-1952.

4. Chaudhuri RR, Peters SE, Pleasance SJ, Northen H, Willers C, et al. (2009) Comprehensive identification of *Salmonella enterica* serovar Typhimurium genes required for infection of BALB/c mice. PLoS Pathog 5: e1000529.

5. Stathopoulos C, Hendrixson DR, Thanassi DG, Hultgren SJ, St Geme JW, 3rd, et al. (2000) Secretion of virulence determinants by the general secretory pathway in Gram-negative pathogens: an evolving story. Microbes and Infection 2: 1061-1072.

6. Kelly A, Goldberg MD, Carroll RK, Danino V, Hinton JC, et al. (2004) A global role for Fis in the transcriptional control of metabolism and type III secretion in *Salmonella enterica* serovar Typhimurium. Microbiology 150: 2037-2053.

7. Gerlach RG, Jackel D, Stecher B, Wagner C, Lupas A, et al. (2007) *Salmonella* Pathogenicity Island 4 encodes a giant non-fimbrial adhesin and the cognate type 1 secretion system. Cell Microbiol 9: 1834-1850.

8. Morgan E, Bowen AJ, Carnell SC, Wallis TS, Stevens MP (2007) SiiE is secreted by the *Salmonella enterica* serovar Typhimurium pathogenicity island 4-encoded secretion system and contributes to intestinal colonization in cattle. Infect Immun 75: 1524-1533.

9. Eckert S, Dziva F, Chaudhuri RR, Langridge GC, Turner DJ, et al. (2011) Retrospective application of transposon-directed insertion-site sequencing to a library of signature-tagged mini-Tn5Km2 mutants of Escherichia coli O157:H7 screened in cattle. J Bacteriol.

10. Lawrence JG, Ochman H (1997) Amelioration of bacterial genomes: rates of change and exchange. J Mol Evol 44: 383-397.

11. Navarre WW, Porwollik S, Wang Y, McClelland M, Rosen H, et al. (2006) Selective silencing of foreign DNA with low GC content by the H-NS protein in *Salmonella*. Science 313: 236-238.

12. Lucchini S, Rowley G, Goldberg MD, Hurd D, Harrison M, et al. (2006) H-NS mediates the silencing of laterally acquired genes in bacteria. PLoS Pathog 2: e81.

13. Dorman CJ (2007) H-NS, the genome sentinel. Nat Rev Microbiol 5: 157-161.

14. Miller VL, Beer KB, Loomis WP, Olson JA, Miller SI (1992) An unusual pagC::TnphoA mutation leads to an invasion- and virulence-defective phenotype in Salmonellae. Infect Immun 60: 3763-3770.

15. Hoiseth SK, Stocker BA (1981) Aromatic-dependent *Salmonella typhimurium* are non-virulent and effective as live vaccines. Nature 291: 238-239.

16. Jones PW, Collins P, Aitken MM (1988) Passive protection of calves against experimental infection with *Salmonella typhimurium*. Vet Rec 123: 536-541.

17. Rankin JD, Taylor RJ (1966) The estimation of doses of *Salmonella typhimurium* suitable for the experimental production of disease in calves. Vet Rec 78: 706-707.

18. Datsenko KA, Wanner BL (2000) One-step inactivation of chromosomal genes in *Escherichia coli* K-12 using PCR products. Proc Natl Acad Sci U S A 97: 6640-6645.

19. Langridge GC, Phan M-D, Turner DJ, Perkins TT, Parts L, et al. (2009) Simultaneous assay of every *Salmonella* Typhi gene using one million transposon mutants. Genome Research 19: 2308-2316.

20. Wallis TS, Paulin SM, Plested JS, Watson PR, Jones PW (1995) The *Salmonella dublin* virulence plasmid mediates systemic but not enteric phases of salmonellosis in cattle. Infect Immun 63: 2755-2761.

21. Barrow PA (1983) A note on a method to aid sampling populations for characteristics. J Appl Bacteriol 54: 311-312.

22. Quail MA, Kozarewa I, Smith F, Scally A, Stephens PJ, et al. (2008) A large genome center's improvements to the Illumina sequencing system. Nature Methods 5: 1005-1010.

23. Hamady M, Walker JJ, Harris JK, Gold NJ, Knight R (2008) Error-correcting barcoded primers for pyrosequencing hundreds of samples in multiplex. Nat Methods 5: 235-237.

24. Kroger C, Dillon SC, Cameron AD, Papenfort K, Sivasankaran SK, et al. (2012) The transcriptional landscape and small RNAs of Salmonella enterica serovar Typhimurium. Proceedings of the National Academy of Sciences of the United States of America 109: E1277-1286.

25. Richardson EJ, Limaye B, Inamdar H, Datta A, Manjari KS, et al. (2011) Genome sequences of Salmonella enterica serovar typhimurium, Choleraesuis, Dublin, and Gallinarum strains of well- defined virulence in food-producing animals. J Bacteriol 193: 3162-3163.

26. R Development Core Team (2008) R: A language and environment for statistical computing. Vienna, Austria: R Foundation for Statistical Computing.

27. Anders S, Huber W (2010) Differential expression analysis for sequence count data. Genome Biol 11: R106.

28. Benjamini Y, Hochberg Y (1995) Controlling the false discovery rate - a practical and powerful approach to multiple testing. Journal of the Royal Statistical Society Series B-Methodological 57: 289-300.

29. Wang Q, Mariconda S, Suzuki A, McClelland M, Harshey RM (2006) Uncovering a large set of genes that affect surface motility in Salmonella enterica serovar Typhimurium. J Bacteriol 188: 7981-7984.

30. Peters SE, Paterson GK, Bandularatne ES, Northen HC, Pleasance S, et al. (2010) *Salmonella enterica* serovar Typhimurium *trxA* mutants are protective against virulent challenge and induce less inflammation than the live-attenuated vaccine strain SL3261. Infect Immun 78: 326-336.

31. Chan RK, Botstein D, Watanabe T, Ogata Y (1972) Specialized transduction of tetracycline resistance by phage P22 in Salmonella typhimurium. II. Properties of a high-frequency-transducing lysate. Virology 50: 883-898.

32. Gal-Mor O, Gibson DL, Baluta D, Vallance BA, Finlay BB (2008) A novel secretion pathway of Salmonella enterica acts as an antivirulence modulator during salmonellosis. PLoS Pathog 4: e1000036.
